# Intravenous synthetic platelet (SynthoPlate) nanoconstructs reduce bleeding and improve ‘golden hour’ survival in a porcine model of traumatic arterial hemorrhage

**DOI:** 10.1038/s41598-018-21384-z

**Published:** 2018-02-15

**Authors:** DaShawn A. Hickman, Christa L. Pawlowski, Andrew Shevitz, Norman F. Luc, Ann Kim, Aditya Girish, Joyann Marks, Simi Ganjoo, Stephanie Huang, Edward Niedoba, Ujjal D. S. Sekhon, Michael Sun, Mitchell Dyer, Matthew D. Neal, Vikram S. Kashyap, Anirban Sen Gupta

**Affiliations:** 1 Department of Pathology, Case Western Reserve University, Cleveland, OH 44106 USA; 2 Department of Biomedical Engineering, Case Western Reserve University, Cleveland, OH 44106 USA; 3 University Hospitals of Cleveland, Division of Vascular Surgery, Cleveland, OH 44106 USA; 4 Department of Surgery, University of Pittsburgh Medical Center, Pittsburgh, PA, 15213 USA

## Abstract

Traumatic non-compressible hemorrhage is a leading cause of civilian and military mortality and its treatment requires massive transfusion of blood components, especially platelets. However, in austere civilian and battlefield locations, access to platelets is highly challenging due to limited supply and portability, high risk of bacterial contamination and short shelf-life. To resolve this, we have developed an I.V.-administrable ‘synthetic platelet’ nanoconstruct (SynthoPlate), that can mimic and amplify body’s natural hemostatic mechanisms specifically at the bleeding site while maintaining systemic safety. Previously we have reported the detailed biochemical and hemostatic characterization of SynthoPlate in a non-trauma tail-bleeding model in mice. Building on this, here we sought to evaluate the hemostatic ability of SynthoPlate in emergency administration within the ‘golden hour’ following traumatic hemorrhagic injury in the femoral artery, in a pig model. We first characterized the storage stability and post-sterilization biofunctionality of SynthoPlate *in vitro*. The nanoconstructs were then I.V.-administered to pigs and their systemic safety and biodistribution were characterized. Subsequently we demonstrated that, following femoral artery injury, bolus administration of SynthoPlate could reduce blood loss, stabilize blood pressure and significantly improve survival. Our results indicate substantial promise of SynthoPlate as a viable platelet surrogate for emergency management of traumatic bleeding.

## Introduction

In remote civilian locations and austere battlefield conditions, uncontrolled traumatic hemorrhage remains one of the leading causes of mortality^1–7^. In such scenarios, transfusion of whole blood or blood components (RBCs, plasma and platelets) can significantly improve survival^8–10^. Extensive trauma resuscitation studies have indicated substantial benefits of early platelet transfusion to treat hemorrhagic shock and enhance survival possibilities^8–13^, however, the limited availability and portability, need for blood type matching, special storage requirements, high risks of bacterial contamination at room temperature and very short shelf-life (3–5 days at room temperature) of platelets, present severe logistical challenges for their applicability in pre-hospital scenarios^14–24^. These issues have led to robust research efforts in improving the storage stability, portability and availability of platelets, e.g. via lyophilization and cold-storage, as well as, developing pathogen reduction technologies to reduce contamination^25–32^. However, these approaches have only marginally improved the pre-hospital applicability of platelets, and the research continues to find alternative platelet-based options, e.g. Isolated Platelet Membrane (IPM Cyplex) and Thrombosome^33–35^. These technologies involve isolation, purification, sterilization, temperature-stabilization and lyophilization of outdated platelets or platelet-derived membrane to utilize their residual hemostatic bioactivity. However, such processing can lead to significant ‘loss-in-function’ of hemostatically relevant glycoprotein on the platelet membrane surface^36,37^, which can potentially lead to batch-to-batch functional inconsistencies. Due to such challenges with natural platelet based products, a parallel area of research has focused on the development of synthetic platelet surrogates^38–40^. The design requirements for such platelet surrogates are: (i) large scale *in vitro* manufacturability, (ii) sterilizability without compromising biofunctional properties, (iii) long-term storage as suspension or lyophilized powder, (iv) easy portability, (v) intravenously administrable ‘on demand’ in pre-hospital scenarios, (vi) post-administration circulation safety without systemic risks, (vii) ability to mimic and amplify endogenous hemostatic mechanisms selectively at the bleeding site, and (viii) biodegradation and safe elimination from the body. Also, such synthetic platelet surrogates can potentially avoid the need for blood type matching and can be used more universally, since they would not bear blood antigens.

**Figure 1:**
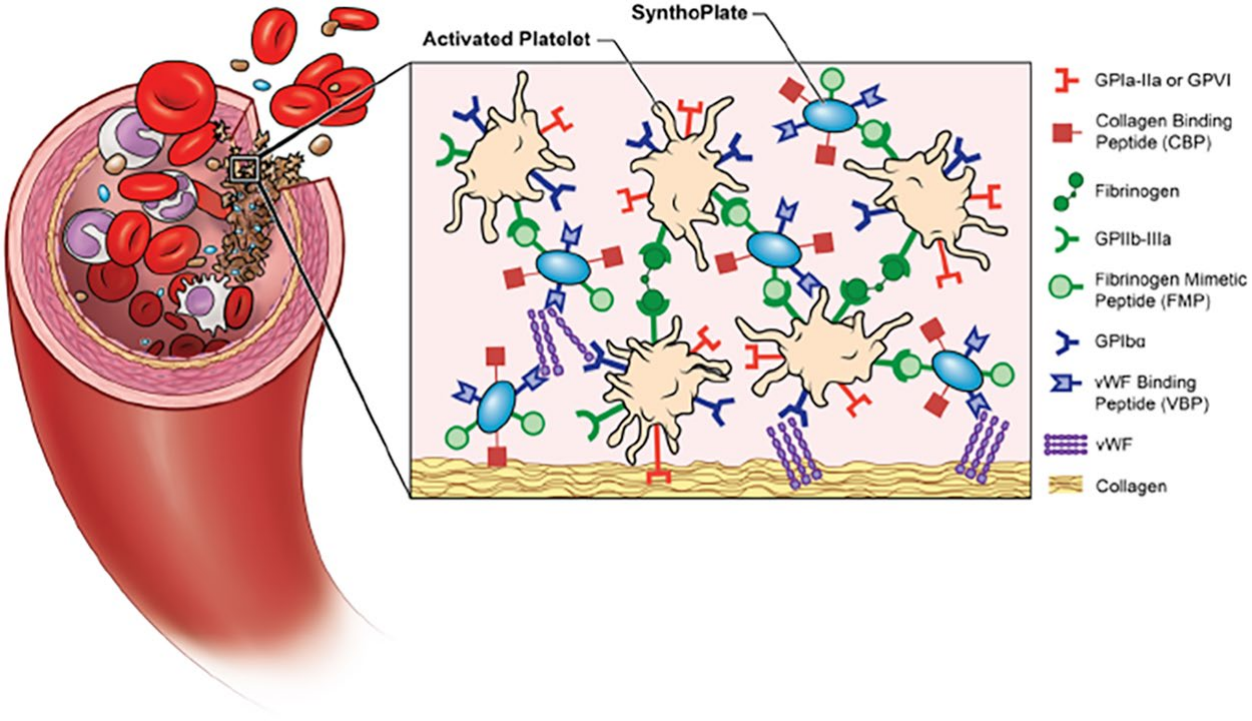
Schematic representation of SynthoPlate design and mechanism, showing nanoconstructs heteromutlivalently decorated with VBP, CBP and FMP motifs to render platelet-mimetic interactions with vWF, collagen and active platelet integrin GPIIb-IIIa respectively, and thus amplify platelet-mediated primary hemostatic mechanisms at the injury site.

As comprehensively reviewed elsewhere^39–41^ and summarily discussed later in this article, several ‘synthetic platelet’ designs have been reported in the past that primarily mimic platelet’s aggregatory capability only via surface-decoration of polymeric, lipidic or albumin particles with fibrinogen (Fg) or Fg-relevant peptides. While these designs have shown some promise as intravenous hemostats, the majority of them have only been reportedly evaluated in small animal bleeding models and only in a pre-treatment framework, i.e. particle administration before bleeding injury. No synthetic platelet design has yet been reported regarding evaluation in a large animal model, that is also in an emergency/rescue framework (i.e. particle administration after bleeding injury) in traumatic bleeding. We have developed a synthetic platelet surrogate, SynthoPlate, by leveraging a clinically relevant biocompatible liposomal platform that is heteromultivalently surface-decorated with three types of peptides: von Willebrand Factor (vWF)-binding peptide (VBP) and collagen-binding peptide (CBP) to mimic platelet’s bleeding site-selective *adhesion* mechanisms and active platelet integrin GPIIb-IIIa-binding fibrinogen-mimetic peptide (FMP) to mimic activated platelet *aggregation* mechanism (Fig. 1)^42–44^. This integrative biomimetic approach to combine platelets’ *adhesion* and *aggregation* functions on a single particle platform is highly unique and can also be adapted to other particle platforms beyond the SynthoPlate’s liposomal platform^45^. The chemical components and manufacturing details for SynthoPlate are provided in the Methods section and the relevant chemical synthesis schemes are shown in Supplementary Figure S.1. We have previously reported the detailed biofunctional and mechanistic characterization of SynthoPlate nanoconstructs^46^ and have also demonstrated that the heteromultivalent integration of platelet-inspired *adhesion* and *aggregation* mechanisms on the same particle platform results in higher hemostatic capability compared to designs bearing ‘*adhesion* only’ or ‘*aggregation* only’ functions^43,45^. We further demonstrated that prophylactic administration of SynthoPlate in normal as well as thrombocytopenic mice could augment hemostatic capability in a non-trauma bleeding model (bleeding time reduction in murine tail transection)^43,45^. Building on these studies, here we sought to investigate the translational promise and hemostatic capability of SynthoPlate in emergency management of traumatic exsanguinating hemorrhage in a pilot scale study in large animal (pig) model. For this, we first characterized the effect of long-term storage (in saline) on SynthoPlate stability, as well as, the effect of sterilization on SynthoPlate stability and biofunction *in vitro*. Next, for *in vivo* studies, we adapted a femoral artery uncontrolled hemorrhage model in pigs, that has been utilized for evaluation of hemostatic dressings^47,48^. In traumatic exsanguinating hemorrhage, significant number of patients succumb within the first 1–2 hours, and hemostatic intervention within this window (known as the ‘golden hour’) can significantly improve survival^10,49,50^. Our model results in the loss of ~1 liter of blood within 15–20 minutes, resulting in 75% mortality within first 45 min. Therefore, we utilized this model to test the SynthoPlate technology I.V.-administered immediately following injury, and the animals were observed for up to 2 hours to monitor blood loss, blood pressure, heart rate and survival. The systemic safety and biodistribution of SynthoPlate was also characterized. Administrations of saline or of unmodified (control) particles were used as comparison groups.

## Results

### SynthoPlate stability in storage

**Figure 2:**
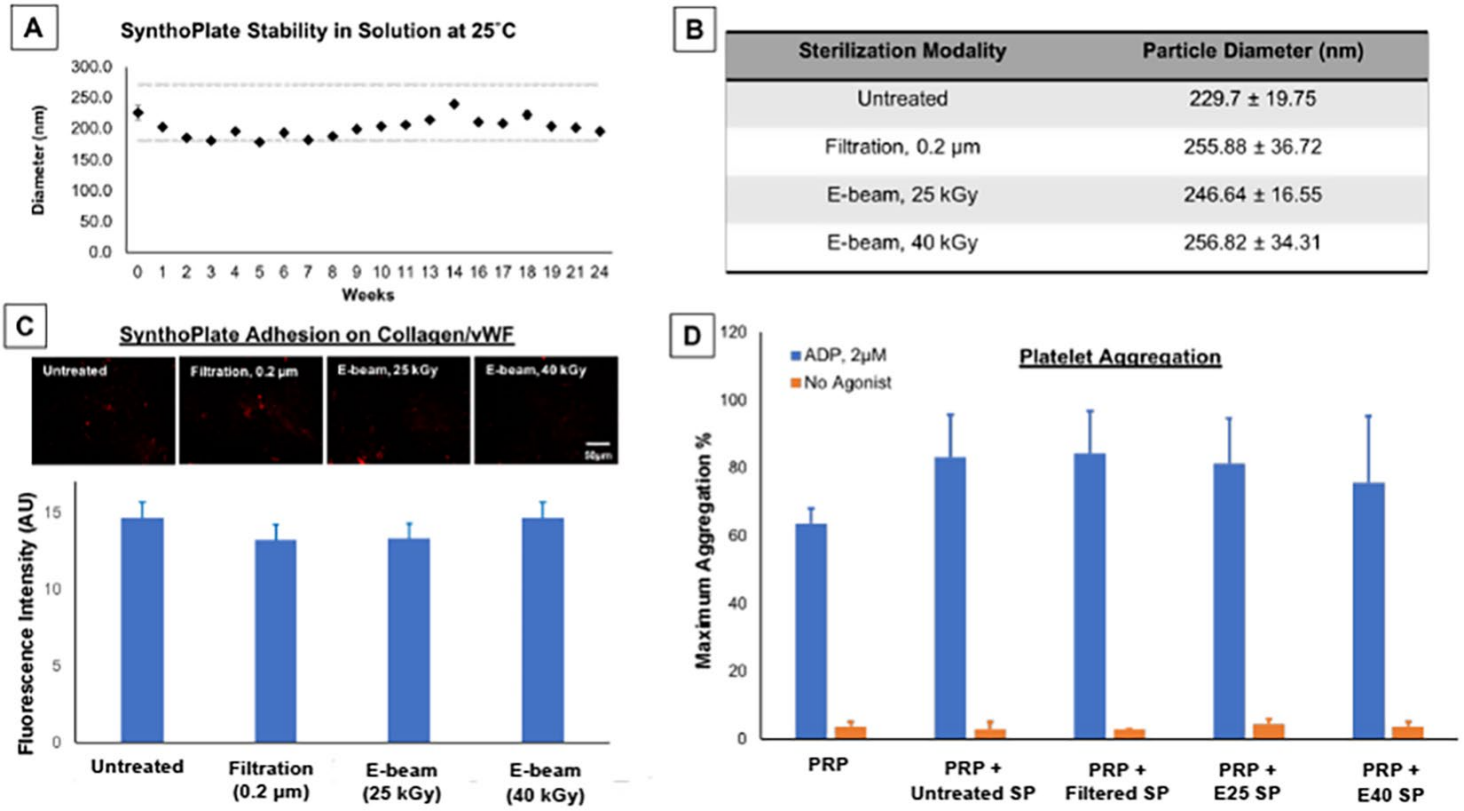
[**A**] SynthoPlate diameter (size distribution) analysis over a 6-month period demonstrated that the particles retained their size within 20% of their starting diameter when stored in a 0.9% NaCl solution at 25 °C, indicating long term stability; [**B**] After sterilization with filtration or E-beam, SynthoPlate showed minimal alteration in size (diameter) compared to fresh-made (unsterilized) samples, indicating that the sterilization did not affect particle stability; [**C**] Representative fluorescent images and quantitative analysis of surface-averaged fluorescence intensity of adhered SynthoPlate showing that sterilized SynthoPlate (red) maintained its ability to adhere to ‘collagen + vWF’-coated surface at levels similar to fresh-made (unsterilized) SynthoPlate, indicating that sterilization did not affect the biofunctional properties of VBP and CBP; [**D**] Aggregometry analysis showed that neither fresh-made (unsterilized) or sterilized SynthoPlate induced spontaneous aggregation of platelets without agonist (w/o ADP), but both unsterilized and sterilized SynthoPlate markedly increased platelet aggregation in the presence of ADP, indicating that sterilization did not affect the biofunctional property of FMP on SynthoPlate and also SynthoPlate did not have any thrombotic risk towards resting platelets.

Liposomal nanoconstruct instability is reflected by large alterations in size (diameter) stemming from particle disassembly, aggregation and clustering. Hence Dynamic Light Scattering (DLS) analysis was used to monitor SynthoPlate stability in saline suspension over a 6-month period. Fresh-made liposome batches show a size (diameter) variability of +/− 15% in laboratory scale manufacture. As shown in Fig. 2A, our analysis showed that when stored as a suspension in 0.9% NaCl at room temperature, SynthoPlate particles showed additional size variability over time of about +/− 5%, thus maintaining their size within an overall variability of +/− 20% of their starting diameter for up to 6 months. Representative DLS data at Month 1, Month 3 and Month 6 are shown in Supplementary Figure S.2. Also, by visual inspection, there were no signs of particle aggregation or settlement out of solution. This suggests that SynthoPlate is amenable to long-term storage under these conditions and indicates their potential for pre-hospital availability as a small volume suspension without compromising stability.

### SynthoPlate sterilization and its effects

SynthoPlate sterilization studies by filtration (0.2 μm filter) as well as E-beam exposure (25 and 40 kGy) were carried out with 6 different challenge organisms, namely *Staphylococcus aureus, Kocuria rhizophila, Clostridium sporogenes, Bacillus subtilis spizizenii, Candida albicans* and *Aspergillus brasiliensis*, as described in the Methods section. For all organisms tested, SynthoPlate itself did not have any bacteriostatic or fungistatic activity, and this data is shown in Supplementary Figure S.3. SynthoPlate solutions sterilized by either filtration or by E-beam exposure showed no visible growth of microorganisms, suggesting effective sterilization by all methods tested. The endotoxin bioburden of the concentrated SynthoPlate formulation (1 × 10^5^ moles lipid per ml) was measured to be 3.5–5 EU/ml as per a standard chromogenic limulous ameobocyte lysate (LAL) assay (see Methods for details). When diluted in sterile saline to the *in vivo* dosing level of 5 × 10^4^ moles lipid per ml, this equates to a dose of ~0.83 EU/kg, which is substantially less than the endotoxin safety threshold of ≤5 EU/kg for *in vivo* usage^51^. Therefore, our SynthoPlate preparation can be considered safe for *in vivo* usage. Furthermore, after sterilization by either filtration or E-beam, SynthoPlate particles showed statistically insignificant variability in size compared to fresh-made (unsterilized) SynthoPlate (Fig. 2B), suggesting that the sterilization methods did not affect particle integrity, size and stability.

To test whether sterilization affects the platelet-mimetic adhesive function of CBP and VBP moieties on SynthoPlate, Rhodamine B-labeled (red fluorescent) SynthoPlate particles in saline suspension, unsterilized or sterilized, were incubated on ‘vWF + collagen’ coated glass coverslips in a 12-well plate with stirring at 120 rpm, following which the coverslips were washed and imaged (experimental details provided in Methods section). Representative images of Rhodamine B (red) fluorescent adhered SynthoPlate, as well as, statistical data of fluorescence intensity analysis from multiple images per condition (5 images per condition), are shown in Fig. 2C. As evident from the results, sterilized SynthoPlate particles retained their capability to adhere to ‘collagen + vWF’-coated surfaces at levels similar to freshly prepared SynthoPlate particles, indicating that sterilization by filtration or E-beam does not affect the platelet-mimetic adhesive activity of VBP and CBP moieties on SynthoPlate. To test whether sterilization affects the ability of FMP moieties on SynthoPlate to interact with active platelet integrin GPIIb-IIIa for amplifying platelet aggregation, lumi-aggregometry assays were performed using platelet rich plasma (PRP) isolated from citrated human whole blood. These assays were performed with 100% PRP or PRP diluted 50% (v/v) with platelet poor plasma (PPP) supplemented with fresh made (unsterilized) or sterilized (filtration and E-beam) SynthoPlate particles, with or without addition of platelet agonist ADP (experimental details in Methods section). In these studies, neither unsterilized nor sterilized SynthoPlate particles were found to induce any spontaneous platelet aggregation in the absence of ADP, but both were able to markedly enhance platelet aggregation in the presence of ADP, as shown in Fig. 2D. Representative raw data from aggregometry studies are shown in Supplementary Figure S.4. Therefore, these results indicate that sterilization by filtration or E-beam does not affect the platelet-mimetic aggregatory activity of FMP moieties on SynthoPlate. Hence, altogether these results establish that SynthoPlate can be effectively sterilized without affecting its platelet-mimetic bioactivity, which is important in the context of its future translation through large-scale manufacturing and sterilization without compromising its hemostatic function.

### SynthoPlate cytotoxicity analysis

SynthoPlate was incubated with 3T3 human fibroblasts (see Methods for culture conditions and viability analysis) at doses relevant to the pig dose used in the current studies (0.5 nM) and double the dose (1 nM). Cell viability was analyzed by standard MTT based metabolic assay and compared to cell cultures without SynthoPlate incubation (i.e. culture media only). As shown in Supplementary Figure S.5. the cell metabolic activity was not affected by SynthoPlate incubation (i.e. no statistical difference), indicating that there is no cytotoxicity associated with SynthoPlate at the dose (as well as double the dose) used *in vivo* in the current studies.

### *In vivo* Safety and Biodistribution Studies in Pigs

**Figure 3:**
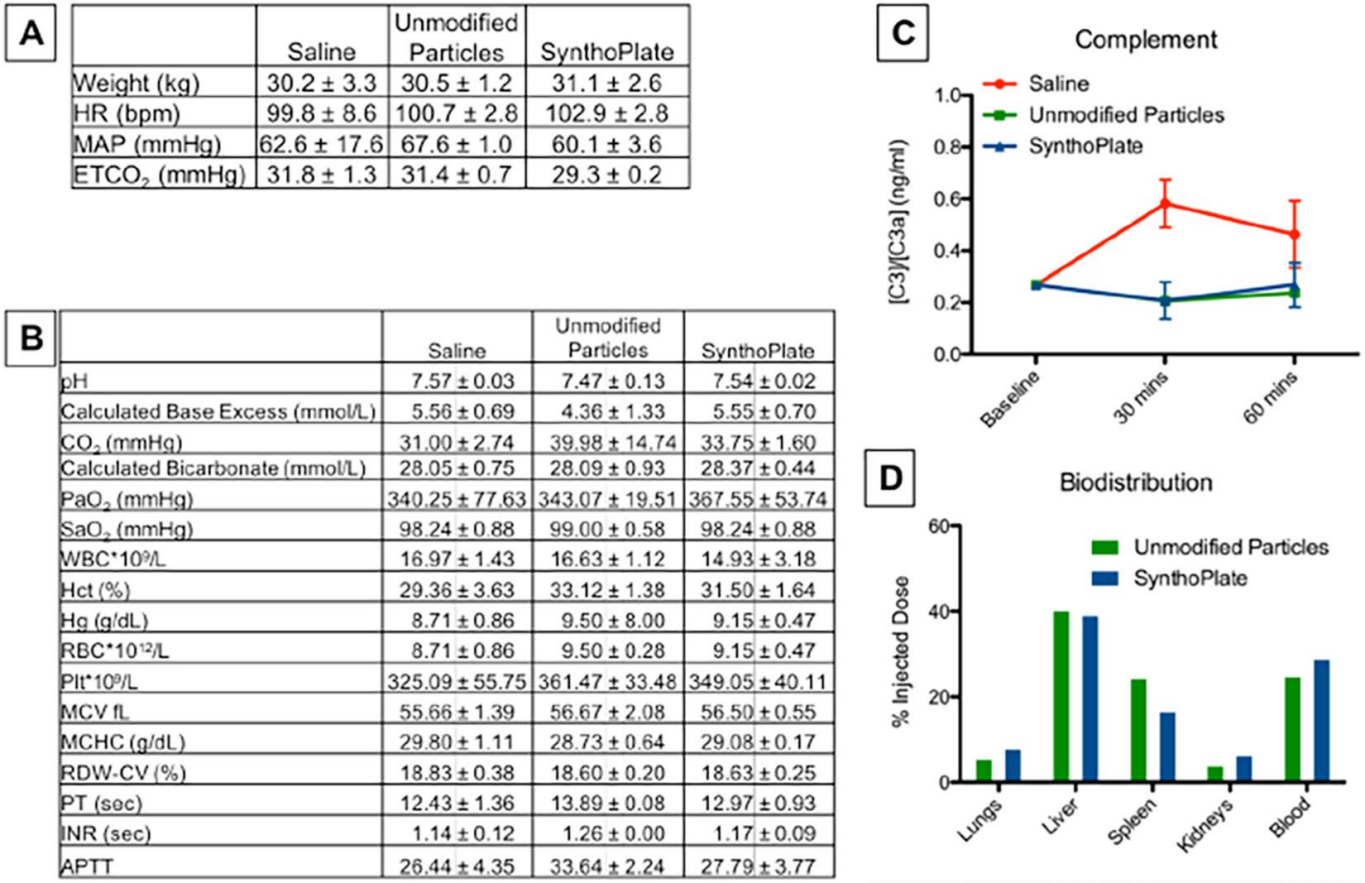
[**A**] Average vitals over the course of the experiment of uninjured pigs and [**B**] average blood lab values over the course of the experiment of uninjured pigs upon administration of unmodified particles or SynthoPlate, showed that there are no statistically significant differences in values compared to saline administration, indicating that SynthoPlate administration has no detrimental physiological effect; [**C**] Analysis of C3:C3a plasma concentration ratios in blood drawn from pigs administered with unmodified particles or SynthoPlate showed no statistically significant alterations of this ratio compared to saline administration groups, indicating that in the dose and administration protocol used, SynthoPlate did not have complement activation (and CARPA) risk; [**D**] Biodistribution analysis from harvested organs indicated a similar distribution profile for unmodified particles and SynthoPlate, with a majority of particles cleared through the liver.

**Figure 4:**
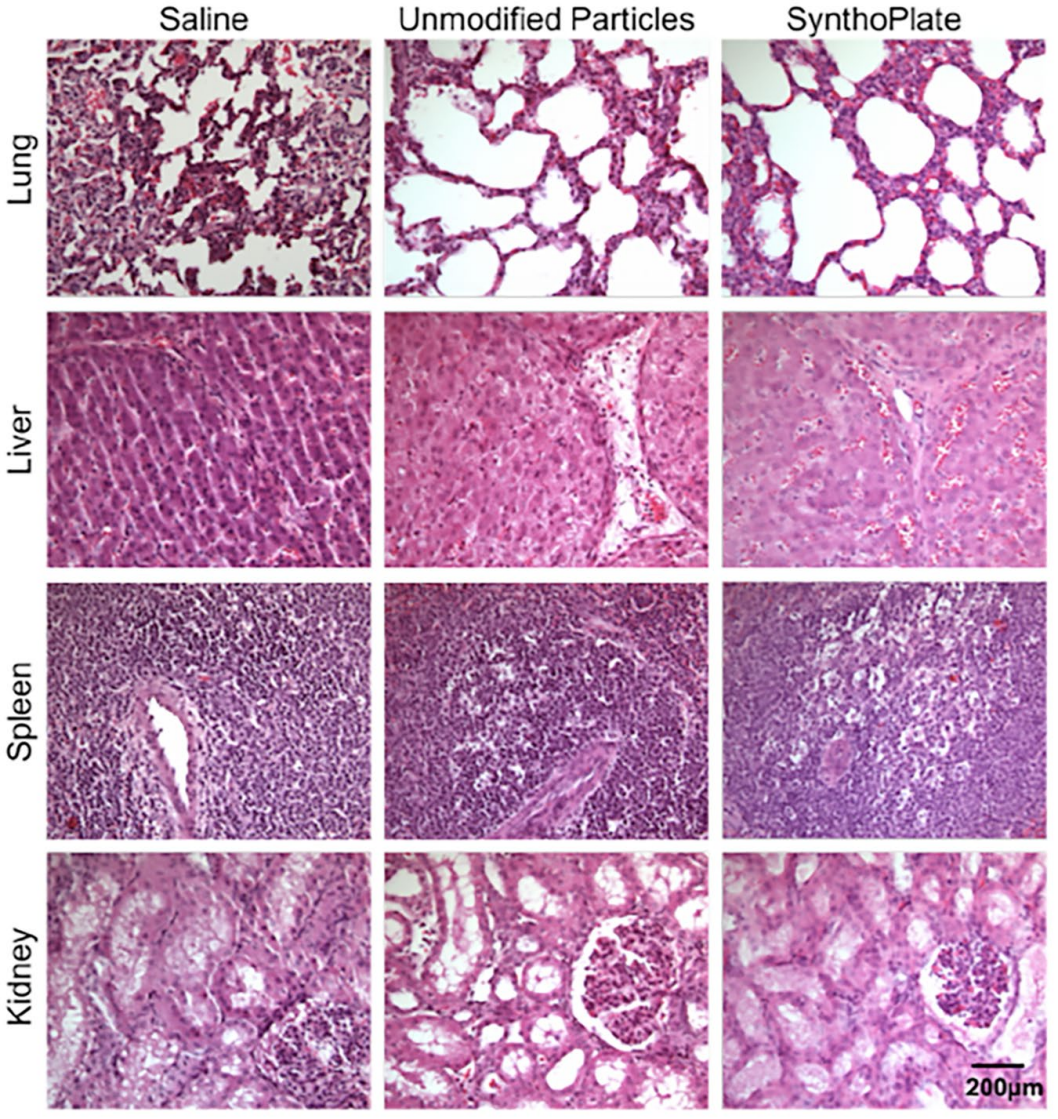
Representative hemotoxylin and eosin (H&E) stained histology images (32× magnification) of organ samples from pigs treated with saline, unmodified particles or SynthoPlate particles, show that SynthoPlate (as well as unmodified particles) does not cause any thrombotic risks in organs; scale bar of 200 μm shown in bottom row kidney image is applicable to all histology images.

The systemic safety of SynthoPlate in pigs within the ‘golden hour’ (60 min) time-frame, was tested by administering the SynthoPlate dose (dosage calculation shown in Supplementary Section S.6) in un-injured pigs intravenously through the jugular vein. Administrations of saline or unmodified (control) particle dose were used as comparison. Two animals per treatment group (i.e. 6 total) were used for these studies. The effects of the treatments on vitals, blood chemistry, complement (C3 to C3a) activation, platelet function (aggregation) and blood clot viscoelastic properties (using ROTEM) were examined for the 60-minute post-administration period (experimental details in Methods section). Sixty minutes post treatment administration, pigs were euthanized and major organs (lungs, heart, liver, kidney, spleen) were harvested for histological (using H&E stain) and biodistribution (using UPLC on tissue homogenate) analysis. As shown in Fig. 3A, following administration of unmodified particles or SynthoPlate in uninjured pigs, none of the animals showed any substantial alteration in their vitals compared to saline. *Ex vivo* analysis of arterial blood collected from the pigs post treatment administration showed no significant changes in the blood analysis parameters between saline and the two particle treatment groups (Fig. 3B), indicating that the SynthoPlate dose did not affect physiological parameters of blood *in vivo*. Also, as shown in Fig. 3C, no significant alterations in the C3:C3a plasma concentration ratios were found in animals administered with unmodified particles or SynthoPlate. It is important to note here that complement ratio alterations of 4-fold or greater is reportedly considered to be a risk indicator for drastic activation of complement system and deleterious reactions like complement activation-related pseudoallergy (CARPA)^52–54^. SynthoPlate (as well as control particles) did not show any such effect compared to saline. Therefore, altogether these results suggest that within our experimental dose and animal observation window, administration of SynthoPlate did not cause any alarming systemic risks. The biodistribution analysis of SynthoPlate (as well as unmodified particles) in uninjured pigs is shown in Fig. 3D, demonstrating that both types of particles have similar organ distribution profile during the 1-hour circulation period. Within this window, most particles of either type were found to be cleared to the liver, possibly by the reticulo-endothelial system (RES), as this mechanism is known for clearance of PEG-ylated liposomes^55^ (base particle for the design of SynthoPlate nanoconstructs). Interestingly ~30% of the injected dose of particles was found to still remain in the systemic circulation (blood), suggesting that this amount can be potentially still available after the 1-hour period to keep localizing at the injury site for hemostatic action. H&E staining-based histopathological analysis of harvested organs from SynthoPlate-injected un-injured animals showed no signs of off-target thrombi in the tissue samples (Fig. 4). Combining these findings with our previous report that SynthoPlate does not activate (and aggregate) resting circulating platelets and also does not spontaneously trigger thrombin (and fibrin) generation in plasma^43,45^, we rationalize that our SynthoPlate dose was safe in pigs, with minimal systemic pro-thrombotic, pro-coagulant and complement activation risks.

### Hemostatic Efficacy Evaluation in Femoral Artery Bleeding Model

**Figure 5:**
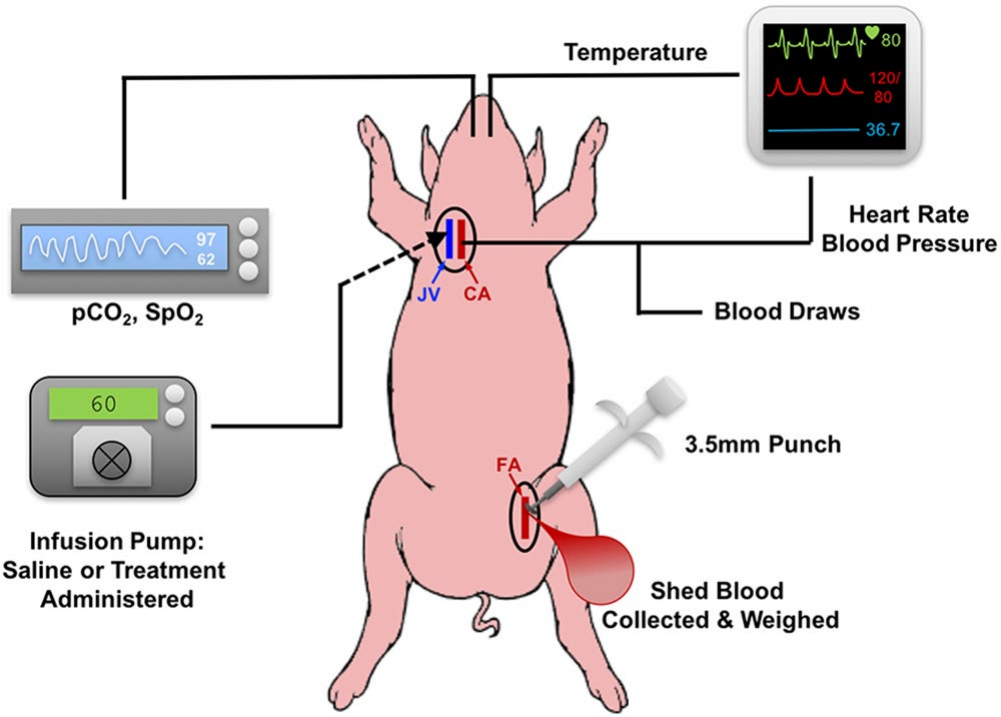
Schematic representation of pig femoral artery hemorrhage model setup. A CO_2_ sensor was placed at the end of the endotracheal tube and mechanical ventilation was provided, EKG electrodes were placed on the pig’s limbs, a pulse-oximeter probe was placed on the pig’s mouth, an esophageal temperature probe was placed to measure core temperature, an angiocatheter was placed in the carotid artery to acquire invasive blood pressure and also withdraw blood samples for *ex vivo* analysis, an angiocatheter was placed in the internal jugular vein to deliver saline (or nanoparticle treatments) via an infusion pump.

**Figure 6:**
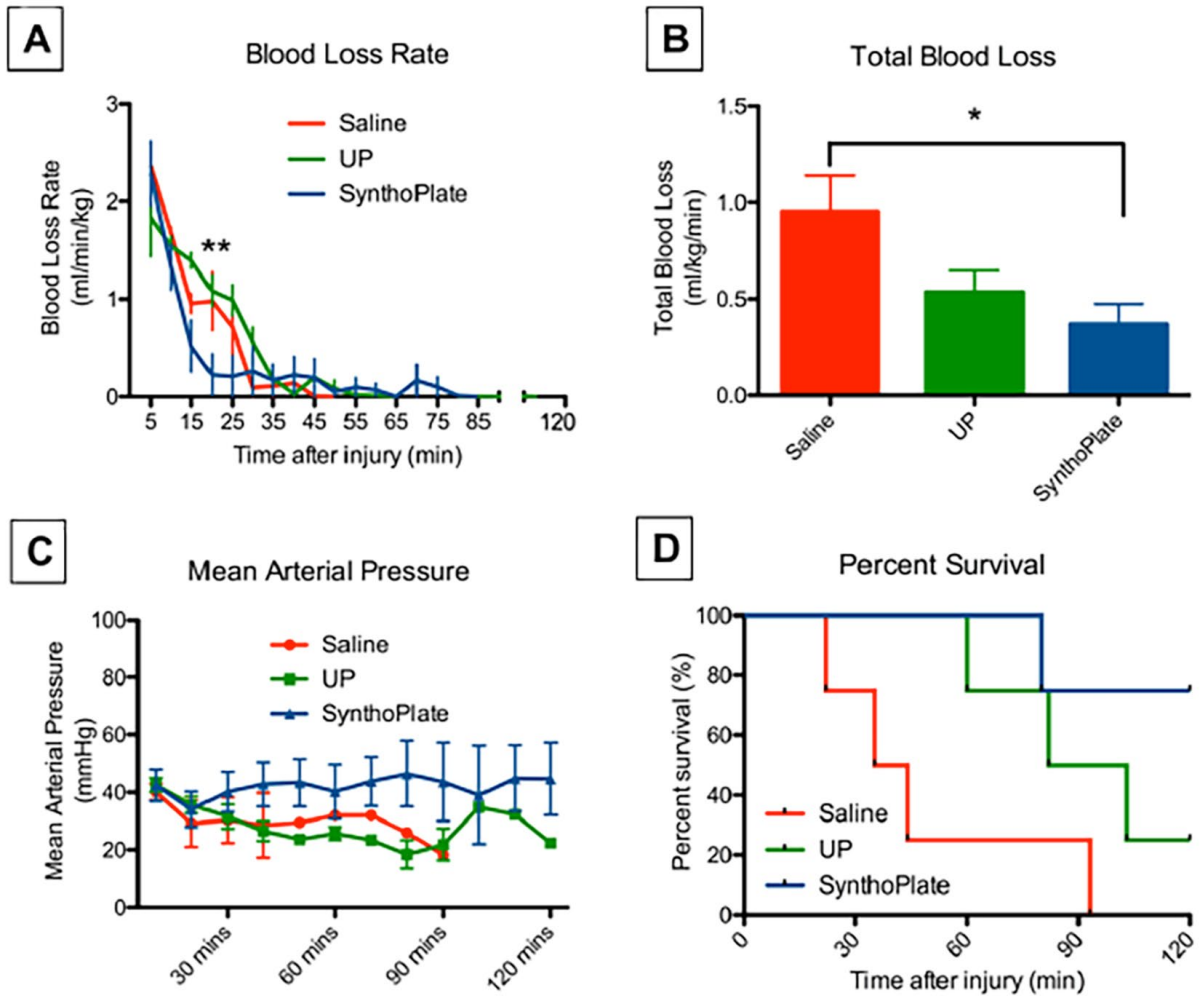
Hemostatic efficacy analysis in injured pigs shows that [**A**] pigs administered with SynthoPlate had a reduced blood loss rate (blue) compared to those treated with saline (red) and unmodified particles (UP, green), especially within first 30 min post treatment administration, where blood loss rate in SynthoPlate treated pig was significantly lower than those treated with unmodified (control) particles and saline (**p < 0.01); [**B**] SynthoPlate administration in pigs also resulted in significantly lower total blood loss compared to saline administration (*p < 0.05); [**C**] SynthoPlate administration in pigs resulted in maintenance and stabilization of a higher average mean arterial blood pressure (MAP) over time (data points shown for every 10 min); [**D**] SynthoPlate administration in pigs resulted in a significant enhancement of survival, with 100% surviving the first 60 min (golden hour) and 75% surviving the additional 60 min (p < 0.05), compared to administration of saline (25% survival by 60 min and 0% survival by ~90 min) or unmodified particles (50% survival by 90 min, 25% survival at 120 min).

**Figure 7:**
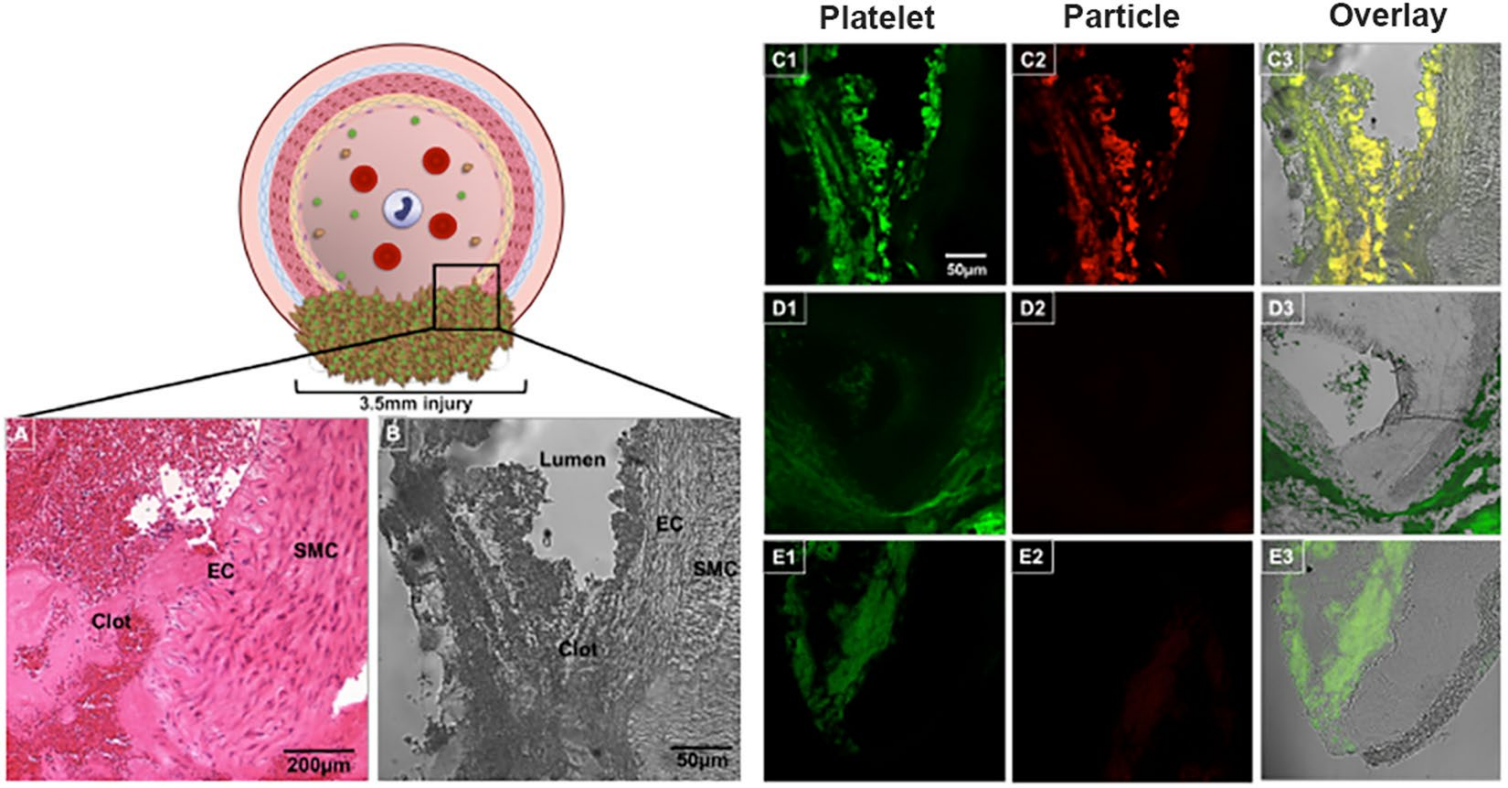
Schematic representation and representative images of hemostasized injury site in the femoral artery of pigs treated with SynthoPlate or saline or control particles: [**A**] Representative hematoxylin and eosin (H&E) stained histology image (32× magnification) and [**B**] representative bright field image (10× magnification) of the site of injury (transected artery) with the injured vessel components (EC: endothelial cell, SMC: smooth muscle cell) and hemostatic clot in view for injured pig treated with SynthoPlate; [C1] representative fluorescence image (10× magnification) of FITC-anti-CD42b (green fluorescence) stained platelets, [C2] Rhodamine B-labeled (red fluorescence) SynthoPlate particles and [C3] overlay of C1 and C2, in the same field of view as the brightfield image B demonstrating that red fluorescent SynthoPlate is co-localized with green fluorescent platelets at the site of injury within the hemostatic clot; [D1, D2 and D3] are similar representative images for injured pig treated with saline, and [E1, E2 and E3] are similar representative images for injured pig treated with unmodified (control) particles showing that some platelets indeed localize at the injury site to promote hemostasis but presence of control particle is minimal at the site and therefore control particles do not have the ability to augment hemostasis; scale bar of 50 μm shown in C1 is applicable to all fluorescent images.

The pig femoral artery hemorrhage model is described in detail in the Methods section and an experimental schematic is shown in Fig. 5. Briefly, an acute hemorrhagic injury (near transection) was caused in the femoral artery with a 3.5 mm arterial punch and 1 minute following injury, saline, unmodified (control) particles or SynthoPlate particles were administered as a bolus dose through the jugular vein. Four animals per treatment group (i.e. 12 total) were used for these studies. Vitals, blood loss, hemodynamic parameters and survival were monitored in real time for 60 minutes (golden hour) and an additional 60 minutes (i.e. total observation for 120 min). During these observations, blood was also drawn through a carotid angiocatheter to run platelet aggregometry and ROTEM analysis *ex vivo*. At the end of experiments, pigs were euthanized (unless they had already succumbed earlier from their injury), and organs and clot were harvested for histological and fluorescence-based distribution analysis (experimental details in Methods section). As shown in Fig. 6A, bolus administration of SynthoPlate immediately following femoral artery injury, resulted in substantial reduction of the blood loss rate over time. It is important to note here that the condition of the saline-treated pigs rapidly declined after injury as indicated by their mean arterial pressure (Fig. 6C) and 75% of them died before 60 minutes (Fig. 6D); therefore, blood loss rate for this group appears to approach zero after this time point (Fig. 6A). Meanwhile, pigs injected with unmodified (control) particle dose continued to undergo low rates of bleeding due to a delay in destabilization of mean arterial pressure (MAP) (Fig. 6B), but ultimately one of them succumbed while actively bleeding around 60 minutes and two other died around 100 minutes (Fig. 6D), though bleeding stopped in both of them at around 60 minutes possibly due to hemorrhage-induced hypotension (Fig. 6A and C). SynthoPlate –treated pigs exhibited significantly lower blood loss rates than comparison groups during the first 30 min and achieved full hemostasis (at 15 min, 18 min, 30 min and 80 min for the 4 pigs respectively) such that the blood loss rate for this group also became zero by the 80-minute time point (Fig. 6A). Furthermore, these animals exhibited stabilized MAP (Fig. 6C) throughout the 120-minute period. Total blood loss for SynthoPlate-treated pigs (normalized to body weight and time survived) was also found to be significantly lower to comparison treatment (Fig. 6B). As shown in Fig. 6D, SynthoPlate-treated animals showed a significantly improved survival, with 100% surviving the golden hour (first 60 minutes) and 75% maintaining survival at the end of 120 minutes. In comparison, 75% of the saline-treated animals died within the first 60 min and 75% of the unmodified (control) particle-treated animals progressively died during the 60–120 min period. Immunohistochemistry and fluorescence imaging analysis of harvested clots from the hemostasized femoral artery of SynthoPlate-treated animals revealed significant incorporation of SynthoPlate in the platelet-rich clot at the site of injury (yellow overlay of green platelets and red SynthoPlate), but no such red fluorescent particle presence was found at the arterial injury site for unmodified (control) particle-treated pigs (representative fluorescence images for the three treatments shown in Fig. 7). Also, similar to the safety experiments in uninjured pigs, during the golden hour (60 min) there were no drastic changes in the C3:C3a plasma concentration for SynthoPlate-treated or unmodified particle-treated animals compared to saline-treated animals (Supplemental Figure S.7.A). Furthermore, the biodistribution of the unmodified particles and SynthoPlate (analyzed at the point of pig death or euthanasia) were similar (Supplemental Figure S.7.B). Aggregometry studies showed that pigs treated with SynthoPlate had a stable platelet aggregation profile while pigs treated with unmodified (control) particle demonstrated a trend for decreasing platelet aggregation and pigs treated with saline showed a trend for platelet hyper-aggregation (Supplemental Figure S.8.A). ROTEM analysis of blood revealed no presence of coagulopathy in SynthoPlate or saline treated animals, as indicated by clotting time (CT), maximum clot firmness (MCF), and amplitude 10 minutes after CT (A10) values from EXTEM and FIBTEM assays that all fall within ‘normal’ reference range, based on criteria established in previous studies (Supplemental Figure S.8.B)^56^. Unmodified particle treated animals did begin to show signs of coagulopathy at the 120-minute time point as indicated by MCF and A10 EXTEM and FIBTEM values that are below the reference range (Supplemental Figure S.8.B). Altogether, the data suggest that SynthoPlate has the ability to reduce blood loss rate as well as total blood loss (Fig. 6A and B), stabilize MAP (Fig. 6C) and increase overall survival (Fig. 6D) after traumatic hemorrhage injury, due to the ability of SynthoPlate to enhance active platelet recruitment and aggregation to form hemostatic clot at the injury site and prevent coagulopathy.

## Discussion

Traumatic exsanguinating hemorrhage continues to be a major cause of military and civilian fatalities in pre-hospital settings, where blood components have limited availability^15,24^. In such scenarios, volume resuscitation with saline or plasma expanders shows only limited benefit^57^. Currently, studies are being directed at improving the survivability in such conditions by anti-fibrinolytic agents like tranexamic acid (TXA) as well as by improving blood component storage, portability and availability through cold-storage and lyophilization^27,58–63^. Time is critical in mitigation of exsanguinating hemorrhage since significant mortalities occur within 1–2 hours of injury, and hemorrhage control within this time can significantly improve survival^49,50^. Therefore, intravenously transfusable synthetic RBC and platelet surrogates that can be administered early at point-of-injury can have tremendous benefit in emergency mitigation of hemorrhagic shock^39–41^.

In the area of synthetic platelet surrogates, past approaches have focused mostly on mimicking only the *aggregatory* mechanism of platelets by decorating micro- and nanoparticles with fibrinogen or fibrinogen-relevant peptides, essentially creating a ‘super-fibrinogen’ construct^39,41^. However, platelet’s native hemostatic action is a co-operative concert of injury site-selective *adhesion* and *aggregation* mechanisms^64^. Hence in our design, we hypothesized that integrating these two mechanisms on a single particle platform will allow a superior mimicry of platelet’s hemostatic mechanisms selectively at the bleeding site while minimizing off-target effects. We previously tested this hypothesis via heteromultivalent decoration of liposomal as well as albumin-based nanoparticles with VBP, CBP and FMP motifs and successfully validated the superior hemostatic efficacy of this integrative design compared to ‘*adhesion* only’ and ‘*aggregation* only’ designs^43,45^. SynthoPlate is a refined liposome-based version stemming from this integrative design^44^, and we have recently demonstrated its hemostatic efficacy in a non-trauma bleeding model in severely thrombocytopenic mice^46^. Building on this, here we investigated the translational promise of the SynthoPlate technology *in vitro* (sterilization effects and storage stability) and *in vivo* (large animal hemorrhage model). Our studies demonstrate that sterilization of SynthoPlate by either filtration or E-beam does not affect the product’s stability and platelet-mimetic biofunctionalities. Our studies also indicate that long-term storage in suspension does not affect SynthoPlate stability. Altogether, these data suggest that sterile suspensions of SynthoPlate can potentially allow long-term storage and availability for hemorrhage mitigation in pre-hospital settings.

Our *in vitro* and *in vivo* studies clearly demonstrate that the SynthoPlate dose did not have any cytotoxicity and systemic risks in the experimental conditions tested. Future studies will be directed at assessing dose escalation based pharmacology and toxicology analysis, as well as, evaluating the potential of multiple doses. Following arterial injury and bolus administration of SynthoPlate (or control) particles, our results show that compared to saline (0% survival), SynthoPlate treatment renders 100% survival during the ‘golden hour’. Interestingly, unmodified (control) particles showed ~50% survival during this first hour, however, survival for this treatment group fell to 25% during the second hour of the experimental window. This can be attributed to the fact that the unmodified particles seemed to transiently improve MAP, possibly due the colloidal nature of injected liposome suspension transiently increasing blood colloidal osmotic pressure (BCOP). However, since unmodified particles were incapable of biofunctionally enhancing hemostasis, these animals continued to bleed at a higher rate and succumbed. In contrast, a combination of hemodynamic stabilization and hemostatic enhancement significantly improved the survival of the SynthoPlate-treated pigs within and beyond the ‘golden hour’. It is to be noted that, because of MAP stabilization and longer survival time, the blood loss rate data (Fig. 6A) apparently suggests that the SynthoPlate-treated animals continued to bleed (albeit at a very low rate) up to and beyond the ‘golden hour’. However, this is a ‘survival bias’, since majority of injured animals treated with saline or unmodified particles essentially could not recover from being rapidly hypotensive, which resulted in ‘apparent reduction’ of blood loss rate and ultimately death. Interestingly, several ‘externally applicable’ hemostatic dressings were recently evaluated in a pig femoral artery hemorrhage model, that showed overall survival to be 50–60% in the 1-hour period^47^. Considering that in our studies, administering only a single bolus intravenous dose of SynthoPlate, with no additional dressings, resulted in 100% survival of the pigs for 60 minutes (and 75% for 120 min), we rationalize that the superior hemostatic capability of SynthoPlate can be potentially combined with externally applied hemostatic dressings, to further improve the hemostatic outcomes in pre-hospital scenarios. We also note that in the current studies the treatments were administered within 1 minute after injury and we have not yet evaluated the ‘administration time window’ for SynthoPlate following injury to determine at what point of delayed administration its hemostatic benefit may fail to increase survival. Our future studies will further evaluate these possibilities in porcine trauma models. Our current findings therefore strongly demonstrate the potential of SynthoPlate as a viable synthetic platelet surrogate for point-of-injury management of traumatic hemorrhagic.

## Materials and Methods

### Materials

The lipid components, namely, 1,2-Distearoyl-sn-glycero-3-phosphocholine (DSPC), 1,2-Distearoyl-sn-glycero-3-phosphoethanolamine-N-[methoxy(polyethylene glycol)-1000] (DSPE-mPEG_1000_), 1,2-Distearoyl-sn-glycero-3-phosphoethanolamine-N-[maleimide(polyethylene glycol)-2000] (DSPE-PEG_2000_-Mal) and 1,2-distearoyl-sn-glycero-3-phosphoethanolamine-N-[azido(polyethylene glycol)-2000] (DSPE-PEG_2000_-Azide) were purchased from Avanti Polar Lipids (Alabaster, AL, USA). Rhodamine B-dihexadecanoyl-sn-glycero-3-phosphoethanolamine (DHPE-RhB) was purchased from Invitrogen (Carlsbad, CA, USA). The peptides CTRYLRIHPQSWVHQI (VBP), C[GPO]_7_ (CBP) and cyclo-{Pra}CNPRGD{Tyr(OEt)}RC (FMP) were custom-synthesized by Genscript (Piscataway, NJ, USA). VBP and CBP were conjugated to DSPE-PEG_2000_-Mal via thiol-maleimide coupling^65^ and FMP was conjugated to DSPE-PEG_2000_-azide via copper-catalyzed alkyne-azide cycloaddition (CuCAAC)^66^ (schematics shown in Supplementary Figure S.1), conjugates were purified by dialysis and characterized by MALDI-TOF mass spectrometry. Sterile normal saline solution (0.9% NaCl) was purchased from Baxter (Deerfield, IL, USA). Cholesterol, rat tail type I collagen, copper(II) sulfate (CuSO_4_), Tris(3-hydroxypropyltriazolylmethyl)amine (THPTA), sodium ascorbate, decaethylene glycol monododecyl (C_12_E_10_) and the Mammalian Cell Lysis Kit were from Sigma-Aldrich (Saint Louis, MO, USA). Ethylenediamine Tetraacetic Acid (EDTA), cellulose dialysis tubing (MWCO 2k and 3.5k), phosphate buffered saline (PBS), chloroform, methanol, LAL Chromogenic Endotoxin Quantification kit, Penicillin-Streptomycin (PS), DMEM without phenol red and Vybrant MTT Cell Proliferation Assay Kit were purchased from Fisher Scientific (Pittsburgh, PA, USA). Citrated human whole blood was drawn from healthy, aspirin-refraining donors via venipuncture according to IRB approved protocols. Adenosine di-phosphate (ADP) and soluble calf skin type I collagen were from Bio/Data Corporation (Horsham, PA, USA), and human vWF (FVIII-free) was from Hematologic Technologies (Essex Junction, VT, USA). For rotational thromboelastometry (ROTEM) analysis, all reagents were purchased from ROTEM (Munich, Germany). For *in vivo* studies on pigs, Yorkshire Farm Pigs were purchased from Shoup Investments Ltd. Telazol, isoflurane and pentobarbital were obtained from Patterson Veterinary (Greely, CO, USA). The Beadbug Mircotube Homogenizer was purchased from Benchmark Scientific (Edison, NJ, USA). The Guinea Pig Complement C3 ELISA kit and Human Complement C3a des Arg ELISA kit were from Abcam (Cambridge, MA, USA). Mouse-derived embryonic fibroblast cell line 3T3 was provided graciously by the laboratory of Eben Alsberg, Case Western Reserve University. High glucose Dulbecco’s Modified Eagle Medium (DMEM) and Fetal Bovine Serum (FBS) were from American Type Culture Collection (Manassas, VA, USA).

### Manufacture of SynthoPlate

SynthoPlate was manufactured using ‘film rehydration and extrusion’ technique as described previously^42,44^. Briefly, DSPC, cholesterol, DSPE-PEG_2000_-VBP, DSPE-PEG_2000_-CBP, DSPE-PEG_2000_-FMP and DSPE-mPEG_1000_ were homogeneously mixed at 0.475, 0.45, 0.0125, 0.0125, 0.025 and 0.025 mole fractions, respectively, in 1:1 chloroform:methanol. Solvent was removed via rotary evaporation, and the thin lipid film was rehydrated with normal saline solution (0.9% NaCl) at a concentration of 1 × 10^5^ moles lipid per mL. This lipid suspension was subjected to 10 freeze/thaw cycles and subsequent extrusion through 200 nm pore diameter polycarbonate membrane using a pneumatic extruder (Northern Lipids, Burnaby, Canada) to create heteromultivalently decorated SynthoPlate vesicles. Dynamic light scattering (DLS) and electron microscopy characterization indicated fresh-made vesicles were ~200 nm in diameter.

### Long term Storage-in-Suspension Studies

In order to evaluate the stability of SynthoPlate under storage, particles were manufactured as described above, packaged into polypropylene tubes, purged with nitrogen gas and stored for up to 6 months at room temperature (~25 °C). Particle size distribution was measured weekly via Dynamic Light Scattering (DLS). This data was considered as reflective of particle stability over time, since large variations in size would be indicative of particle instability.

### Studies on SynthoPlate sterilization and its biofunctional effects

SynthoPlate samples were evaluated at Lexamed (Toledo, OH, USA) for sterilization via filtration and E-beam exposure. First, it was necessary to validate the sterilization methods by demonstrating that SynthoPlate itself does not inhibit the growth of common contaminating organisms and hence does not interfere with the standard sterilization assays. For this, SynthoPlate samples were inoculated with 6 different challenge organisms, namely *Staphylococcus aureus, Kocuria rhizophila, Clostridium sporogenes, Bacillus subtilis spizizenii, Candida albicans* and *Aspergillus brasiliensis*, and allowed to grow in appropriate conditions for up to 5 days. Any differences in growth profiles between samples with or without SynthoPlate over the 5-day period were recorded. Next, SynthoPlate samples were sterilized by filtration in 0.2 μm filter and by E-beam exposure at both 25 and 40 kGy doses. Sterility was assessed by observation of the samples for any visible growth of microorganisms over the course of 7 days. Furthermore, endotoxin burden in the formulation was evaluated using a standard chromogenic limulous ameobocyte lysate (LAL) assay. Briefly, nanoparticle samples were incubated with 0.5% (v/v) of 1.5% C_12_E_10_ detergent for 20 minutes at 65 °C followed by 1 hour at room temperature to solubilize the lipid components of SynthoPlate and release any masked endotoxin^67^. Endotoxin burden in the detergent-treated SynthoPlate samples was quantified using the LAL assay.

To assess whether sterilization affects SynthoPlate particle stability, post-sterilization particle size distribution was measured by DLS and compared to fresh-made (unsterilized) particles. Furthermore, in order to measure whether sterilization affects the platelet-mimetic biofunctional abilities of SynthoPlate, specific adhesion and aggregation assays were performed as adapted from our previous reports^43,46^. To test whether sterilization affects the platelet-mimetic adhesive function of CBP and VBP moieties on SynthoPlate (to collagen and vWF, respectively), acid washed glass coverslips were coated with a solution of 1:1 vWF:collagen, and 200 µl RhB-labeled SynthoPlate suspensions (0.4 mg/ml), un-sterilized or sterilized, were incubated on these coverslips in a 12-well plate for 30 minutes at 37 °C on a shaker set at 100 rpm. Following this, SynthoPlate suspension was removed, and loosely bound particles were further washed away with PBS. The coverslips were then mounted onto microscope slides and imaged (using Zeiss AxioObserver inverted fluorescence microscope) for adhered SynthoPlate (Rhodamine B fluorescence, λ_ex_ = 560 nm, λ_em_ = 580 nm). To test whether sterilization affects the ability of FMP moieties to bind to active platelet integrin GPIIb-IIIa to amplify platelet aggregation, platelet lumi-aggregometry assays were performed using a PAP-8E (Bio/Data Corp, Horsham, PA, USA) instrument. For this, human whole blood was collected in citrated tubes from healthy consenting donors in accordance with protocols and guidelines approved by the Institutional Review Board (IRB) of University Hospitals Cleveland Medical Center (UHCMC, IRB Number 12–16–06). Informed consent was obtained from all subjects as per this IRB-approved guideline. The whole blood was centrifuged at 150 × g for 15 minutes to obtain platelet rich plasma (PRP). Some of the PRP was further centrifuged at 2500 × g for 25 minutes to obtain platelet poor plasma (PPP). Aggregometry studies were carried out at 37 °C with stirring at 1200 rpm. ADP-mediated aggregation was assessed with 225 μl of 100% PRP or PRP diluted 50% (v/v) with PPP supplemented with fresh-made (un-sterilized) or sterilized (filtration and E-beam) SynthoPlate particles with a particle concentration of 5 × 10^10^/ mL, with or without adding agonist (2 × 10^−5^ M ADP). These adhesion and aggregation results of SynthoPlate post-sterilization were compared to that for fresh-made (unsterilized) SynthoPlate.

### In vitro cytotoxicity assay with SynthoPlate

3T3 cells were grown in DMEM supplemented with 10% FBS and 1% PS. Cultures were maintained in a humidified atmosphere of 5% CO_2_ at 37 °C. 3T3 cells were seeded into 96 well plates at 5000 cells/well. Within 24 hours, media was removed and seeded wells were treated with media containing SynthoPlate particles versus media without the particles. SynthoPlate particles were applied at 0.5 nM and 1 nM concentrations, which correspond to the dose administered to pigs and double the dose administered to the pigs respectively. All treatments were suspended in phenol red free DMEM and an n = 8 was maintained for all test groups. The treated cells were incubated at normal culture conditions for 24 hours. After 24 hours, a standard MTT (3-(4,5-dimethylthiazol-2-yl)-2,5-diphenyltetrazolium bromide) assay was used to assess metabolic viability of the cells. In this assay, metabolic activity (i.e. cell viability) reduces the MTT dye to insoluble formazan which has a purple color, and the intensity of this can be assessed by colorimetric (i.e. UV-Visible spectrometry) technique to reflect cell viability. For this, post incubation with or without SynthoPlate, media was removed and fresh phenol red free media and MTT stock solution were applied to all wells. The cells were incubated at normal culture conditions for 4 hours. After 4 hours, a solution of SDS-HCl was mixed into all wells. and incubated for a final period of 4 hours. Finally, the formazan intensity (i.e. cell metabolic viability) was quantified with UV-visible spectrometry, measuring absorbance at 570 nm.

### ***In vivo*Safety and Biodistribution Studies in Pigs**

All *in vivo* porcine studies were carried out in accordance with relevant guidelines and regulations approved by Case Western Reserve University Institutional Animal Care and Use Committee (IACUC, Protocol Number 2015-0135). Yorkshire Farm Pigs 25–36 kg were acclimated to the laboratory space for 48 hours. Pigs were fasted for 12 hours before experiments but had free access to water. Pigs were sedated with Telazol (6–8 mg/kg) injected intramuscularly, intubated with an endotracheal tube and anesthetized with isoflurane (1–5% to effect). A CO_2_ sensor was placed at the end of the endotracheal tube. Mechanical ventilation was provided to keep end-tidal CO_2_ and respiration rate initially at normal values. EKG electrodes were placed on the pig’s limbs. A pulse-oximeter probe was placed on the pig’s mouth (cheek or tongue). An esophageal temperature probe was placed to measure core temperature, which was maintained between 36 °C–38 °C with a water-filled warming blanket. An angiocatheter was placed in the carotid artery to acquire invasive blood pressure and withdraw blood samples for *ex vivo* testing. Another angiocatheter was placed in the internal jugular vein to deliver saline or particle treatments. The 50 ml dose of SynthoPlate (or control particles) to be administered in the pigs was 1 × 10^11^ particles/ml (per calculation shown in Supplementary Section S.4) followed by a 450 ml saline infusion. Invasive arterial blood pressure, CO_2_, SpO_2_, temperature, and heart rate were recorded every 30 seconds for the first 10 minutes, every minute for the next 20 minutes, and every 5 minutes thereafter for a total of 60 minutes. Arterial blood was drawn via the carotid artery angiocatheter at baseline, 30 minutes, and 60 minutes. At the end of experiments, pigs were euthanized with an I.V. overdose of pentobarbital (0.22 ml/kg).

In order to evaluate the systemic safety of SynthoPlate in pigs within the ‘golden hour’ time-frame, without any confounding effects from injury, treatments were administered (at the bolus dose stated previously) in a pilot group of non-injured animals (total n = 6; saline: n = 2, unmodified particles: n = 2, SynthoPlate: n = 2), and the animals were observed for 1 hour. The surgeons were blinded to the administered treatments, and blinding was ensured by a researcher who assigned a randomly generated code to each treatment. The effects of the administered treatments on vitals, blood chemistry, platelet function (aggregation), and blood clot viscoelastic properties were examined. It has been reported that nanoparticles (including liposomes) can sometimes lead to a hypersensitivity reaction and complement activation (known as complement activation-related pseudoallergy or CARPA)^52^. Therefore, the risk for such reactions was assessed *ex vivo*. For this, specific complement activation marker (C3 to C3a) was measured in the blood drawn from the pig at baseline as well as 30 and 60 minutes post administration of saline, unmodified particles or SynthoPlate. In complement activation, C3 is cleaved into C3a and C3b leading to a decrease in plasma C3 levels and an increase in plasma C3a levels, and thus the C3:C3a ratio can indicate such risks. For these studies, platelet poor plasma was isolated from pig blood samples by centrifuging for 25 minutes at 2500 × g and measurements of C3 and C3a were carried out using Guinea Pig Complement C3 ELISA kit and Human Complement C3a des Arg ELISA kit respectively (Abcam, Cambridge, MA, USA). Pig whole blood samples were also analyzed for pH, calculated base excess, carbon dioxide (CO_2_), calculated bicarbonate, partial pressure of oxygen (PaO_2_), oxygen saturation (SaO_2_), white blood cell count (WBC), hematocrit (Hct), hemoglobin (Hg), red blood cell count (RBC), platelet count (Plt), mean corpuscular volume in femtoliters (MCV fL), mean corpuscular hemoglobin concentration (MCHC), red blood cell distribution width- coefficient of variation (RDW-CV), prothrombin time (PT), international normalized ratio (INR), and, activated partial thromboplastin time (APTT) compared between blood of saline-treated, unmodified particle-treated and SynthoPlate-treated pigs. Furthermore, platelet aggregometry was conducted on PRP isolated from pig blood samples, using methods described previously. In addition, effect of the various treatment groups on clot viscoelastic properties was assessed by Rotational Thromboelastometry (ROTEM) via analysis of drawn blood samples.

At the 1-hour time point pigs were euthanized and major organs (lungs, heart, liver, kidney, spleen) were harvested, weighed and fixed in formalin. Three random samples 200–350 mg were removed from each pig organ and placed into pre-weighed homogenizing tubes, and the wet weight of each organ sample was recorded. The samples were then vacuum dried and their dry weights recorded. The dry organs were homogenized with a BeadBug Microtube Homogenizer using a Mammalian Cell Lysis kit and then centrifuged (20 minutes, 12,000 × g) to separate the organ tissue from the supernatant. The supernatant was added to 1:1 methanol:chloroform to dis-assemble the Rhodamine B labeled lipids of SynthoPlate in the samples. These samples were analyzed with Ultra Performance Liquid Chromatography (UPLC) using a fluorescence detector (λ_ex_ = 560 nm, λ_em_ = 580 nm) to assess Rhodamine B fluorescence. Biodistribution of the SynthoPlate particles was determined by calculating the nanogram (ng) of particles per milliliter (ml) of supernatant utilizing an appropriate calibration curve that correlates RhB fluorescence intensity to particle concentration. Samples from each organ were also processed for histological analysis. For this, samples were embedded in paraffin and sectioned and stained with hematoxylin and eosin (H & E stain). The resultant slides where imaged on a Zeiss Axiophot microscope and images were obtained with Zeiss Axiovision camera and software.

### Femoral Artery Bleeding Model in Pigs

Figure 5 shows a representative experimental set-up of the model for these studies. Pigs (total n = 12; Saline: n = 4, unmodified particles: n = 4, SynthoPlate: n = 4) were prepared as described previously. The skin at the inguinal area of the thigh was incised ~10 cm, the femoral artery was exposed and injured (near transection) using a 3.5 mm aortic punch (Scanlan International, St Paul, Minnesota, USA). Saline or particle treatments were administered 1 minute after injury via the jugular at a rate of 50 ml/min. Blood loss from femoral artery injury was measured by carefully suctioning the shed blood without disturbing the injury site, into a container that was weighed every 30 seconds for the first 10 minutes, every minute for the next 20 minutes, and every 5 minutes thereafter until bleeding stopped due to death or hemostasis. Blood mass was correlated to blood volume by approximating blood density to be 1000 kg/m^3^. With saline administration, which is a standard volume resuscitation strategy for treating hemorrhage in pre-hospital scenarios, this injury resulted in an average of 31.5 mL/kg blood loss in 24 minutes, and showed only 25% survival within the first 60 min (i.e. within the ‘golden hour’ period). At the end of experiments, pigs were euthanized with an I.V. overdose of pentobarbital (0.22 ml/kg).

### Hemostatic Efficacy Evaluation in Femoral Artery Bleeding Model

The femoral artery bleeding model was performed as described above. Induction of the injury was designated as time-point zero (0). Saline or particle (unmodified control or SynthoPlate) treatments were administered 1 minute after injury via the jugular, (50 ml particle concentration 1 × 10^11^ particles/ml followed by 450 ml saline) at 50 ml per minute. Blood loss was measured as described previously. Arterial blood was drawn via the carotid artery angiocatheter at baseline, and 15, 30, 60 and 120 minutes, to run *ex vivo* analysis. At the end of experiments, pigs were euthanized, major organs (lungs, heart, liver, kidney, spleen) and clot were harvested, weighed and fixed in formalin. Organ samples were prepared and analyzed for SynthoPlate (or control unmodified particle) biodistribution at time of death as described previously for uninjured animals. Sections of the femoral artery injury site from SynthoPlate-treated animals were deparaffinized and rehydrated by washing with xylene 2 times for 2 minutes, 1:1 xylene: ethanol for 3 minutes, ethanol 2 times for 3 minutes, 95% ethanol for 3 minutes, 70% ethanol for 3 minutes and finally 50% ethanol for 3 minutes. Antigen retrieval was performed by incubating in Tris-EDTA buffer in a water bath at 60 °C overnight. Slides were washed, blocked in 10% serum with 1%BSA in TBS for 2 hours, incubated with the FITC-labeled (green fluorescent) CD42b antibody (for staining platelet glycoprotein GPIbα), washed, protected with a cover-slip and then imaged (using a Zeiss AxioObserver inverted fluorescence microscope). Brightfield, SynthoPlate fluorescence (red, Rhodamine B) and platelet fluorescence (green, FITC) were captured for the same field of view.

### Statistical Analysis

Statistical analysis of aggregometry, biodistribution, blood loss data, and complement data was done using two-way ANOVA with a Bonferroni post-test. Vitals data, blood analysis data and ROTEM data were analyzed using one-way ANOVA with a Tukey post-test. Survival data was analyzed using a Log-rank test. In all analyses, significance was considered to be p < 0.05.

### Data Availability

Majority of data generated and analyzed during this study are included in this article (and its Supplementary Information files). Additional raw data (e.g. raw data of size distribution analysis by DLS, H&E staining data of harvested organ sections from all animals, raw data from ROTEM and aggregometry analysis etc.) will be available from the corresponding author on reasonable request.

## Electronic supplementary material

Supplementary Information

## References

[1] Kauvar DS, Lefering R, Wade CE (2006). Impact of Hemorrhage on Trauma Outcome: An Overview of Epidemiology, Clinical Presentations, and Therapeutic Considerations. J Trauma..

[2] Holcomb JB (2007). Causes of Death in U.S. Special Operations Forces in the Global War on Terrorism, 2001–2004. Ann. Surg..

[3] Blackbourne LH (2012). Military medical revolution: prehospital combat casualty care. J. Trauma Acute Care Surg.

[4] Cohen, M.J. *et al*. PROMMTT Study Group: Clinical and mechanistic drivers of acute traumatic coagulopathy. *J. Trauma Acute Care Surg*. **75**, S40–S47 (2013). 10.1097/TA.0b013e31828fa43d PMC3755603 23778510

[5] Dorlac WC (2005). Mortality from isolated civilian penetrating injury. J. Trauma.

[6] Smith ER, Shapiro G, Sarani B (2016). The profile of wounding in civilian public mass shooting fatalities. J. Trauma Acute Care Surg.

[7] van Oostendorp SE, Tan ECTH, Geeraedts LMG (2016). Prehospital control of life-threatening truncal and junctional haemorrhage is the ultimate challenge in optimizing trauma care; a review of treatment options and their applicability in the civilian trauma setting. Scand. J. Trauma Resusc. Emerg. Med.

[8] Holcomb, J. B. *et al*. PROPPR Study Group: Transfusion of plasma, platelets, and red blood cells in a 1:1:1 vs a 1:1:2 ratio and mortality in patients with severe trauma: the PROPPR randomized clinical trial. *JAMA* **313**, 471–482 (2015). 10.1001/jama.2015.12 PMC4374744 25647203

[9] Holcomb, J. B. *et al*. PROMMTT Study Group: The prospective, observational, multicenter, major trauma transfusion (PROMMTT) study: comparative effectiveness of a time-varying treatment with competing risks. *JAMA Surg*. **148**, 127–146 (2013). 10.1001/2013.jamasurg.387 PMC3740072 23560283

[10] Holcomb JB (2007). Damage control resuscitation: directly addressing the early coagulopathy of trauma. J. Trauma.

[11] Borgman MA (2007). The ratio of blood products transfused affects mortality in patients receiving massive transfusions at a combat support hospital. J. Trauma.

[12] Davenport R (2014). Haemorrhage control of the pre-hospital trauma patient. Scand. J. Trauma Resusc. Emerg. Med.

[13] Ishikura H, Kitamura T (2017). Trauma-induced coagulopathy and critical bleeding: the role of plasma and platelet transfusion. J. Intensive Care.

[14] Carmen R (1993). The selection of plastic materials for blood bags. Transf. Med. Rev.

[15] Lambert MP, Sullivan SK, Fuentes R, French DL, Poncz M (2013). Challenges and promises for the development of donor-independent platelet transfusions. Blood.

[16] Moroff G, Holme S (1991). Concepts about current storage conditions for the preparation and storage of platelets. Transf. Med. Rev.

[17] Devine DV, Serrano K (2010). The platelet storage lesion. Clin. Lab. Med..

[18] Bordin JO, Heddle NM, Blajschman MA (1994). Biologic effects of leukocytes present in transfused cellular blood products. Blood.

[19] Heddle NM (1993). A prospective study to identify the risk factors associated with acute reactions to platelet and red cell transfusions. Transfusion.

[20] Blajchman MA (1998). Bacterial contamination and proliferation during the storage of cellular blood products. Vox Sang.

[21] Schreiber GB, Busch MP, Kleinman SH, Korelitz JJ (1996). The risk of transfusion- transmitted viral infections. The Retrovirus Epidemiology Donor Study. N. Engl. J. Med..

[22] Corash L (1998). Inactivation of viruses, bacteria, protozoa, and leukocytes in platelet concentrates: current research perspectives. Transf. Med. Rev.

[23] Milford EM, Reade MC (2016). Comprehensive review of platelet storage methods for use in the treatment of active hemorrhage. Transfusion.

[24] Spinella PC (2012). Constant challenges and evolution of US military transfusion medicine and blood operations in combat. Transfusion.

[25] Hoffmeister KM (2003). Glycosylation restores survival of chilled blood platelets. Science.

[26] Pidcoke HF (2013). Primary hemostatic capacity of whole blood: a comprehensive analysis of pathogen reduction and refrigeration effects over time. Transfusion.

[27] Reddoch KM (2014). Hemostatic function of apheresis platelets stored at 4 °C and 22 °C. Shock.

[28] Noorman F (2016). Transfusion: −80 °C Frozen blood products are safe and effective in military casualty care. PLoS ONE.

[29] Acker, J. P., Marks, D. C. & Sheffield, W. P. Quality assessment of established and emerging blood components for transfusion. *J. Blood Transfus*. **2016**, 10.1155/2016/4860284 (2016). 10.1155/2016/4860284 PMC5192317 28070448

[30] Seghatchian J, de Sousa G (2006). Pathogen-reduction systems for blood components: The current position and future trends. Transfusion and Apheresis Science.

[31] Reddy HL (2008). Toxicity testing of a novel riboflavin-based technology for pathogen reduction and white blood cell inactivation. Transfusion Med. Rev.

[32] Janetzko K, Hinz K, Marschner S, Goodrich R, Klüter H (2009). Pathogen reduction technology (Mirasol) treated single-donor platelets resuspended in a mixture of autologous plasma and PAS. Vox Sang.

[33] Nasiri S (2013). Infusible platelet membrane as a platelet substitute for transfusion: an overview. Blood Transfus.

[34] Fitzpatrick GM, Cliff R, Tandon N (2013). Thrombosomes: a platelet-derived hemostatic agent for control of noncompressible hemorrhage. Transfusion.

[35] Sum R (2007). Wound-healing properties of trehalose-stabilized freeze-dried outdated platelets. Transfusion.

[36] Chao FC (1996). Infusible platelet membrane microvesicles: a potential transfusion substitute for platelets. Transfusion.

[37] Biro E (2005). The phospholipid composition and cholesterol content of platelet-derived microparticles: a comparison with platelet membrane fractions. J. Thromb. Haemost..

[38] Blajchman MA (1999). Substitutes for success. Nature Med..

[39] Modery-Pawlowski CL (2013). Approaches to synthetic platelet analogs. Biomaterials.

[40] Sen Gupta A (2017). Bio-inspired nanomedicine strategies for artificial blood components. Wiley Interdiscip Rev Nanomed Nanobiotechnol.

[41] Hickman DA, Pawlowski CL, Sekhon UDS, Marks J, Sen Gupta A (2017). Biomaterials and advanced technologies for hemostatic management of bleeding. Adv. Mater..

[42] Ravikumar M, Modery CL, Wong TL, Sen Gupta A (2012). Peptide-decorated Liposomes Promote Arrest and Aggregation of Activated Platelets under Flow on Vascular Injury Relevant Protein Surfaces *In Vitro*. Biomacromolecules.

[43] Modery-Pawlowski CL, Tian LL, Ravikumar M, Wong TL, Sen Gupta A (2013). *In vitro* and *in vivo* hemostatic capabilities of a functionally integrated platelet-mimetic liposomal nanoconstruct. Biomaterials.

[44] Sen Gupta, A. & Ravikumar M. Synthetic Platelets. US Patent 9107845 (2015).

[45] Anselmo AC (2014). Platelet-like nanoparticles: mimicking shape, flexibility, and surface biology of platelets to target vascular injuries. ACS Nano.

[46] Shukla M (2017). *In vitro* characterization and *in vivo* evaluation of SynthoPlate (synthetic platelet) technology in severely thrombocytopenic mice. J. Tromb. Haemost.

[47] Rall JM, Cox JM, Songer AG, Cestero RF, Ross JD (2013). Comparison of novel hemostatic dressings with QuikClot combat gauze in a standardized swine model of uncontrolled hemorrhage. J. Trauma Acute Care Surg.

[48] Fülop A, Turóczi Z, Garbaisz D, Harsányi L, Szijártó A (2013). Experimental models of hemorrhagic shock: A review. Eur Surg Res.

[49] Hess JR (2013). Resuscitation of trauma-induced coagulopathy. Hematology Am. Soc. Hematol Educ. Program.

[50] Cowley RA (1975). A total emergency medical system for the state of Maryland. Md State Med. J.

[51] Li Y, Boraschi D (2016). Endotoxin contamination: a key element in the interpretation of nanosafety studies. Nanomedicine (Lond).

[52] Szebeni J (1999). Hemodynamic changes induced by liposomes and liposome-encapsulated hemoglobin in pigs. Circulation.

[53] Szebeni J, Muggia F, Gabizon A, Barenholz Y (2011). Activation of complement by therapeutic liposomes and other lipid excipient-based therapeutic products: Prediction and prevention. Adv. Drug Del. Rev.

[54] Urbanics R, Bedőcs P, Szebeni J (2015). Lessons learned from the porcine CARPA model: constant and variable responses to different nanomedicines and administration protocols. Eur. J. Nanomed.

[55] Immordino ML, Dosio F, Cattel L (2006). Stealth liposomes: review of the basic science, rationale, and clinical applications, existing and potential. Int. J. Nanomed.

[56] Hunt H (2015). Thromboelastography (TEG) and rotational thromboelastometry (ROTEM) for trauma induced coagulopathy in adult trauma patients with bleeding. Cochrane Database Syst. Rev..

[57] Coats TJ, Brazil E, Heron M (2006). The effects of commonly used resuscitation fluids on whole blood coagulation. Emerg. Med. J..

[58] CRASH-2 trial collaborators, *et al*. Effects of tranexamic acid on death, vascular occlusive events, and blood transfusion in trauma patients with significant haemorrhage (CRASH-2): a randomized, placebo-controlled trial. *Lancet* **376**, 23–32 (2010). 10.1016/S0140-6736(10)60835-5 20554319

[59] Brown JB (2015). Design of the Study of Tranexamic Acid during Air Medical Prehospital Transport (STAAMP) Trial: Addressing the Knowledge Gaps. Prehosp. Emerg. Care.

[60] Snyder EL, Rinder HM (2003). Platelet storage – Time to come in from the cold?. N. Engl. J. Med..

[61] Apelseth TO, Cap AP, Spinella PC, Hervig T, Strandenes G (2017). Cold stored platelets in treatment of bleeding. ISBT Science Series.

[62] Cap AP, Perkins JG (2011). Lyophilized platelets: challenges and opportunities. J. Trauma.

[63] Cap AP, Spinella PC (2017). Just chill – it’s worth it!. Transfusion.

[64] Hoffman M, Monroe DM (2001). A cell-based model of hemostasis. Thromb. Haemost..

[65] Elliott JT, Prestwich GD (2000). Maleimide-functionalized lipids that anchor polypeptides to lipid bilayers and membranes. Bioconjugate Chem.

[66] Presolski SI, Hong VP, Finn MG (2011). Copper-catalyzed azide-alkyne click chemistry for bioconjugation. Curr. Protoc. Chem. Biol.

[67] Harmon P (1997). The release and detection of endotoxin from liposomes. Anal. Biochem..

