# Supplementary material for: Intravenous synthetic platelet (SynthoPlate) nanoconstructs reduce bleeding and improve ‘golden hour’ survival in a porcine model of traumatic arterial hemorrhage
Source: Sci Rep. 2018 Feb 15;8:3118. doi: 10.1038/s41598-018-21384-z (PMC5814434; doi:10.1038/s41598-018-21384-z)

**Intravenous synthetic platelet (SynthoPlate) nanoconstructs reduce bleeding and improve ‘golden hour’ survival in a porcine model of traumatic arterial hemorrhage**

DaShawn A Hickman^1^, Christa L Pawlowski^2^, Andrew Shevitz^3^, Norman F Luc^2^, Ann Kim^3^, Aditya Girish^2^, Joyann Marks^2^, Simi Ganjoo^2^, Stephanie Huang^2^, Edward Niedoba^2^, Ujjal D S Sekhon^2^, Michael Sun^2^, Mitchell Dyer^4^, Matthew D Neal^4^, Vikram S Kashyap^3^, Anirban Sen Gupta^2*^

^1^Department of Pathology, Case Western Reserve University, Cleveland, OH 44106, USA

^2^Department of Biomedical Engineering, Case Western Reserve University, Cleveland, OH 44106, USA

^3^University Hospitals of Cleveland, Division of Vascular Surgery, Cleveland, OH 44106, USA

^4^Department of Surgery, University of Pittsburgh Medical Center, Pittsburgh, PA, USA

**Corresponding Author E-mail: anirban.sengupta@case.edu**

**Supplementary Information:**


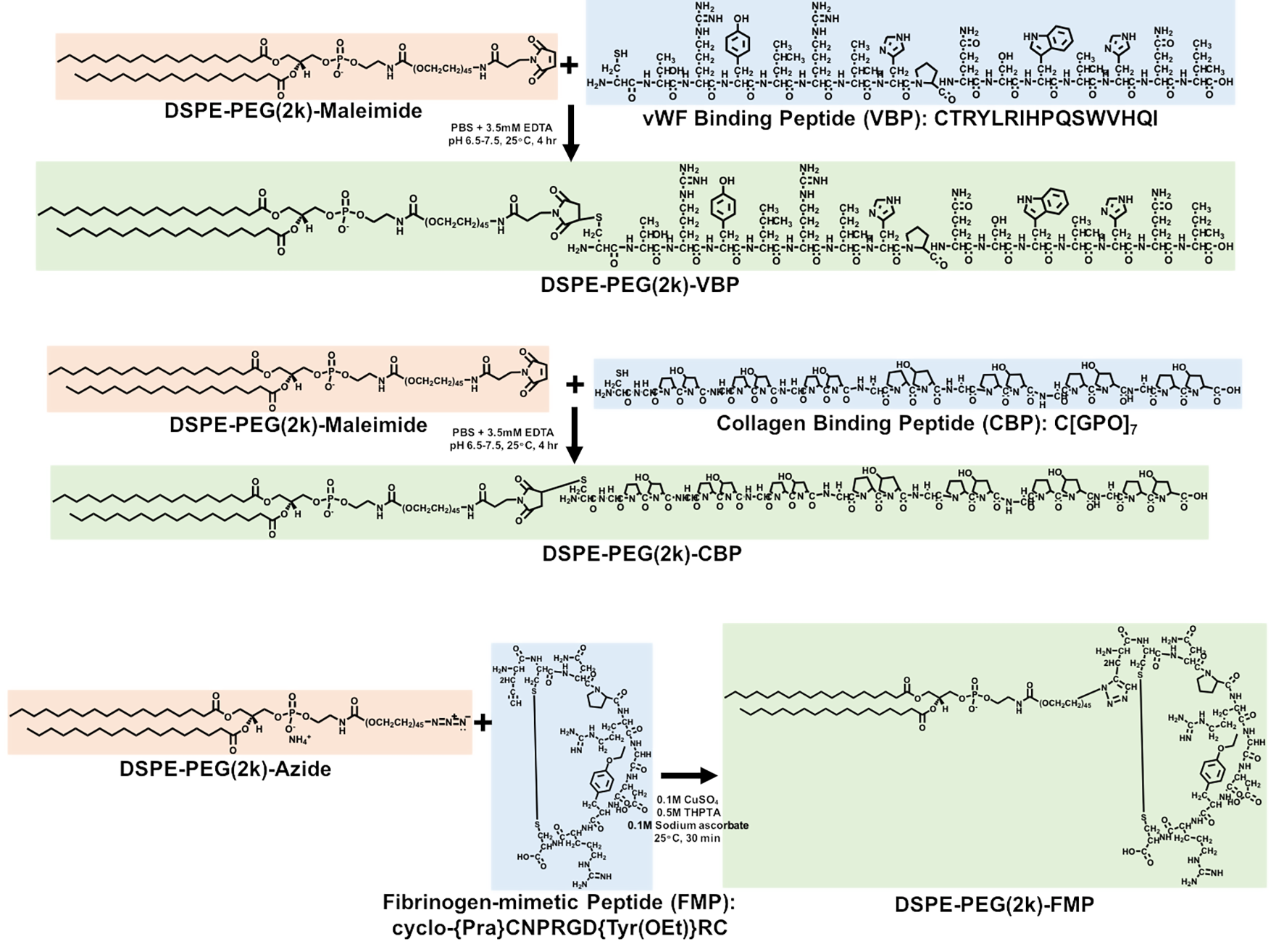


**Figure S.1.** Lipid-to-peptide bioconjugation schemes (along with corresponding chemical structures) for synthesizing DSPE-PEG-VBP, DSPE-PEG-CBP and DSPE-PEG-CMP molecules, that were used to manufacture the SynthoPlate nanoconstruct.


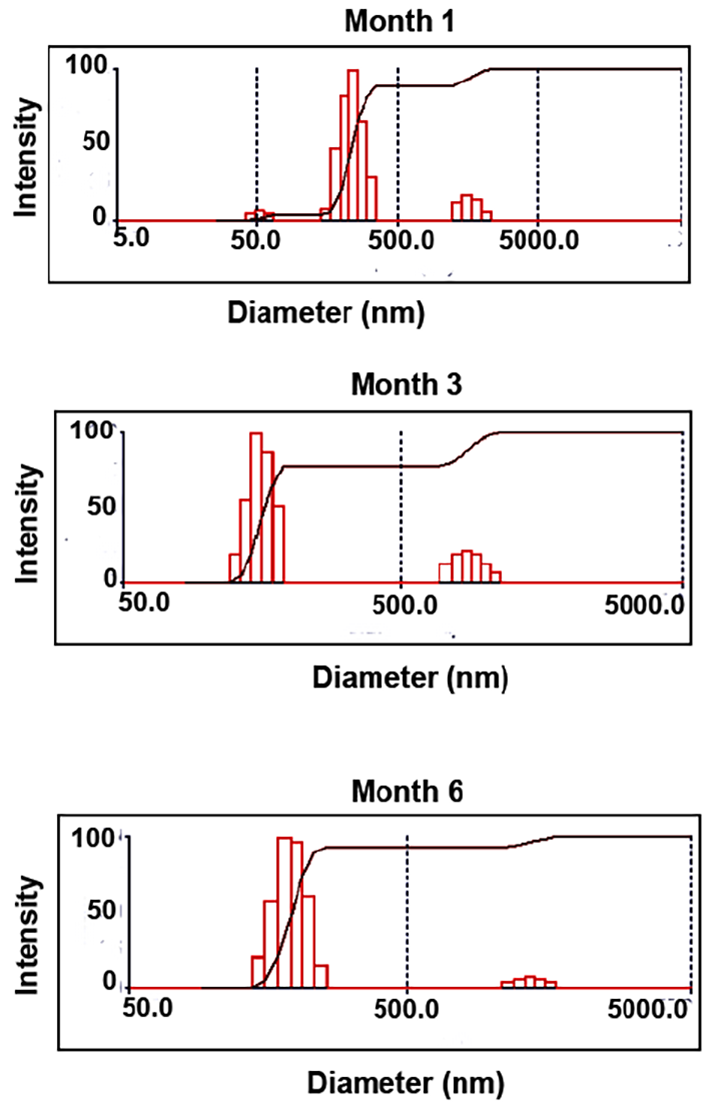


**Figure S.2.** Representative Dynamic Light Scattering (DLS) data of SynthoPlate size distribution characterization over a 6-month period; data is shown for 1 month, 3 month and 6 month time points, demonstrating that there is minimal alteration in particle diameter and therefore indicating particle stability in storage (saline suspension at 25^o^C).

| 25 kGy - LIQUID | |  |  |  |  |  |  |  |  |  |
| --- | --- | --- | --- | --- | --- | --- | --- | --- | --- | --- |
| **Organism** | **Days of Incubation** | | | | | | | | |  |
|  | 1 | | 2 | | 3 | | 4 | | 5 | |
|  | T | C | T | C | T | C | T | C | T | C |
| B. subtilis | N | N | P | P | PE | PE | PE | PE | PE | PE |
| C. albicans | N | N | N | N | P | P | PE | PE | PE | PE |
| A. brasiliensis | N | N | N | N | N | N | P | P | PE | PE |
| S. aureus | N | N | P | P | PE | PE | PE | PE | PE | PE |
| K. rhizophila | N | N | N | N | P | P | PE | PE | PE | PE |
| C. sporogenes | P | P | PE | PE | PE | PE | PE | PE | PE | PE |
|  |  |  |  |  |  |  |  |  |  |  |
| 40 kGy - LIQUID | |  |  |  |  |  |  |  |  |  |
| **Organism** | **Days of Incubation** | | | | | | | | |  |
|  | 1 | | 2 | | 3 | | 4 | | 5 | |
|  | T | C | T | C | T | C | T | C | T | C |
| B. subtilis | N | N | P | P | PE | PE | PE | PE | PE | PE |
| C. albicans | N | N | N | N | P | P | PE | PE | PE | PE |
| A. brasiliensis | N | N | N | N | N | N | P | P | PE | PE |
| S. aureus | N | N | P | P | PE | PE | PE | PE | PE | PE |
| K. rhizophila | N | N | N | N | P | P | PE | PE | PE | PE |
| C. sporogenes | P | P | PE | PE | PE | PE | PE | PE | PE | PE |

**Figure S.3.** Bacteriostatic and fungistatic analysis of SynthoPlate suspension exposed to E-beam sterilization and challenged with 6 different organisms, namely *Bacillus subtilis spizizenii, Candida albicans, Aspergillus brasiliensis, Staphylococcus aureus, Kocuria rhizophila,* and *Clostridium sporogenes,* that were allowed to grow in appropriate conditions for up to 5 days. T= test, C= control, N = No growth, P = positive growth and PE = positive growth established.


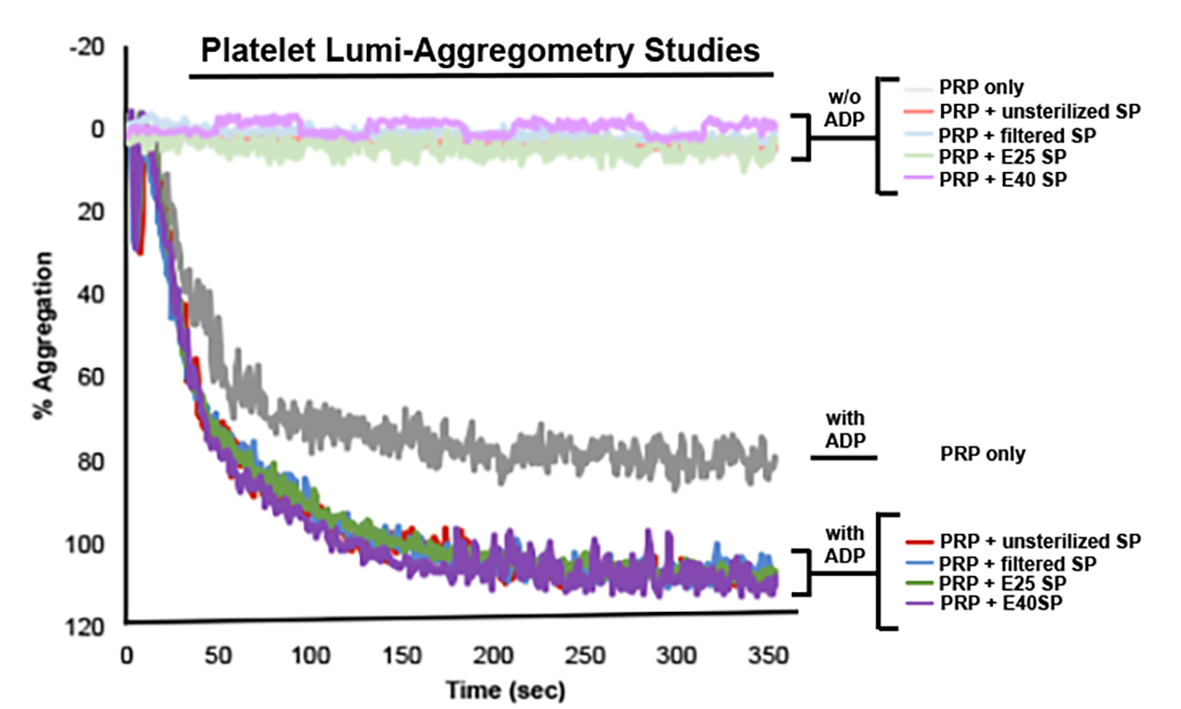


**Figure S.4.** Representative Lumi-Aggregometry raw data from studies on SynthoPlate (SP, fresh unsterilized versus sterilized) interaction with platelets (resting or agonist-activated) in platelet-rich-plasma (PRP); results indicate that neither unsterilized nor sterilized SynthoPlate has any activating and aggregatory effect on resting platelets (top group of traces w/o ADP), while both unsterilized and sterilized SynthoPlate are capable of enhancing the aggregation of activated platelets (bottom group of traces with ADP) above that seen for PRP only. These results also demonstrate that sterilization does not affect the pro-aggregatory function of SynthoPlate on activated platelets.

**
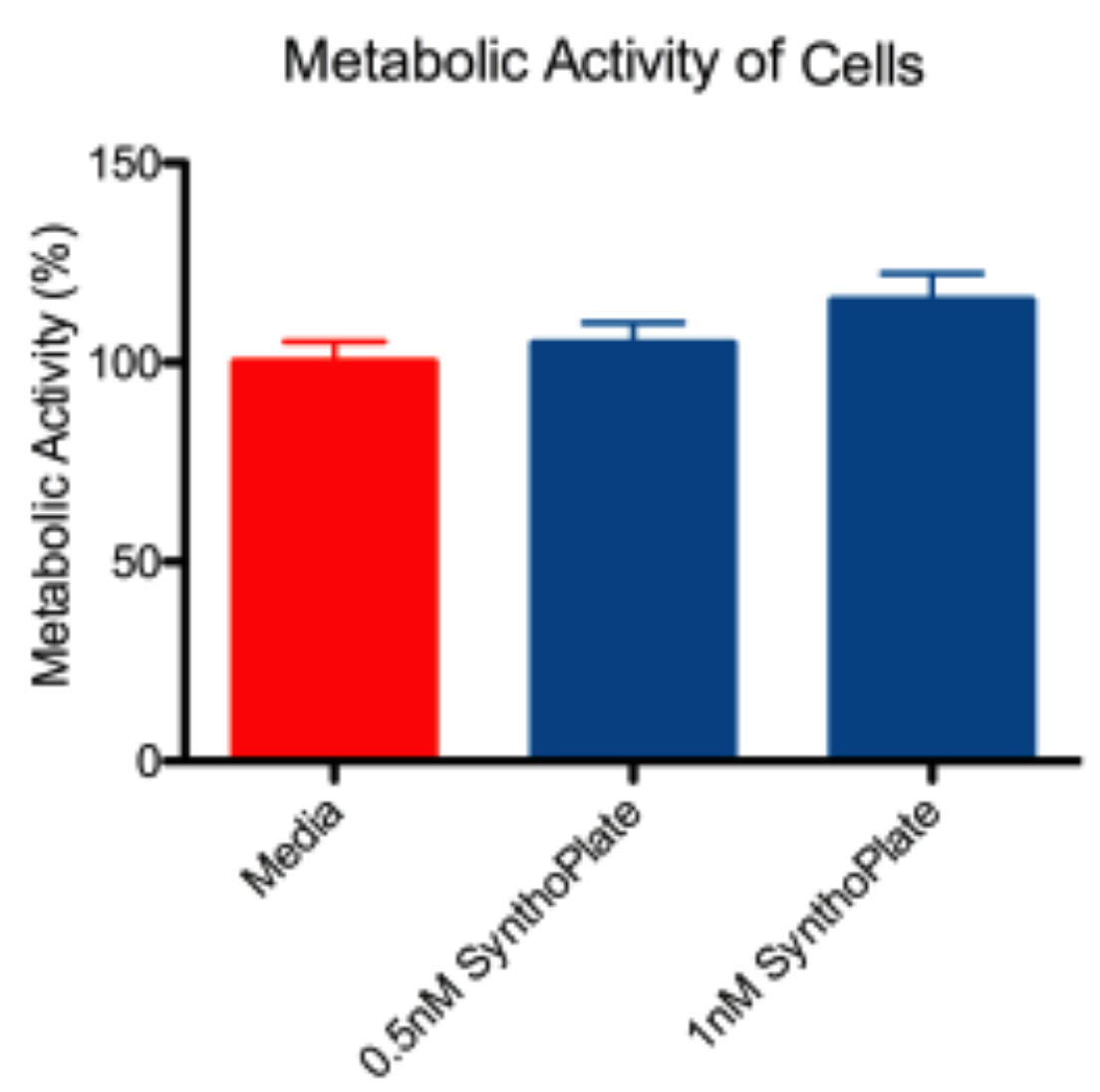
**

**Figure S.5.** MTT-based metabolic activity analysis data of human 3T3 fibroblasts in culture incubated with 0.5 nM SynthoPlate (dose relevant to in vivo studies) or 1 nM SynthoPlate (double of in vivo dose) showed no statistical difference compared to cell metabolic activity in culture media only, indicating that SynthoPlate is not cytotoxic at the dose used.

**Supplemental Section S6.** In vivo SynthoPlate dose calculation: A typical unit of platelets for human transfusion contains ≥ 3 x 10^11^ platelets. Since actual platelets are ~10 times larger (in diameter) than SynthoPlate, we decided to scale the transfusion of SynthoPlate (or control particles) by 1 order of magnitude, to 5 x 10^12^ particles per dose. Dose calculation:

$$5*{10}^{12} particles per dose\div50 ml saline bolus volume=1*{10}^{11} particles/ml$$

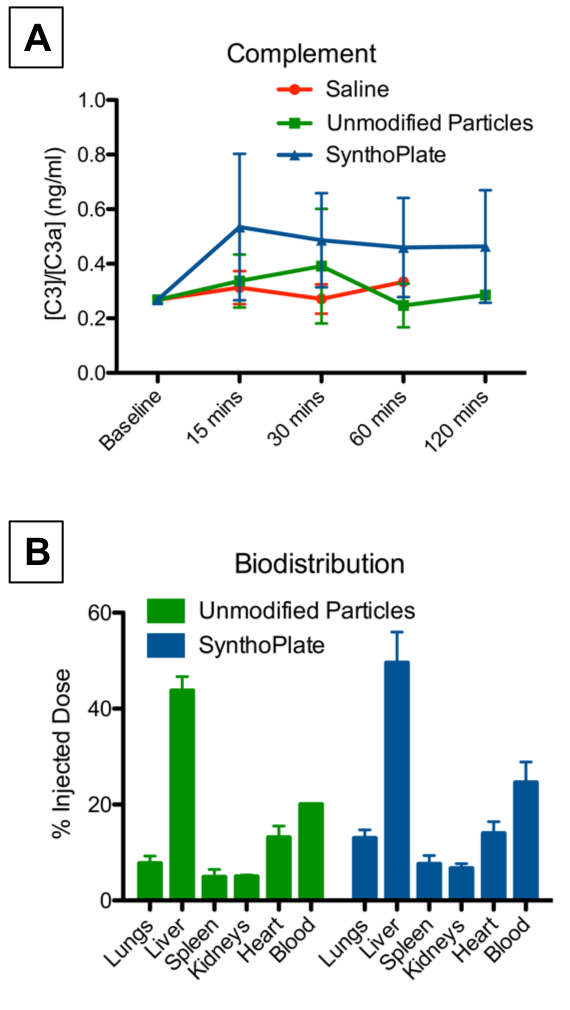


**Figure S.7.** [A] Complement (C3:C3a) analysis data on drawn blood from pigs and [B] biodistribution data from pigs subjected to femoral artery injury and administered intravenously with unmodified particles or SynthoPlate nanoconstructs.

**Figure S.8.** [A] Representative ex vivo Lumi-aggregometry analysis data and [B] ROTEM analysis data (CT, MCF and A10 parameters) of blood samples drawn from pigs after being subjected to femoral artery injury and administered intravenously with saline or unmodified particles or SynthoPlate nanoconstructs.


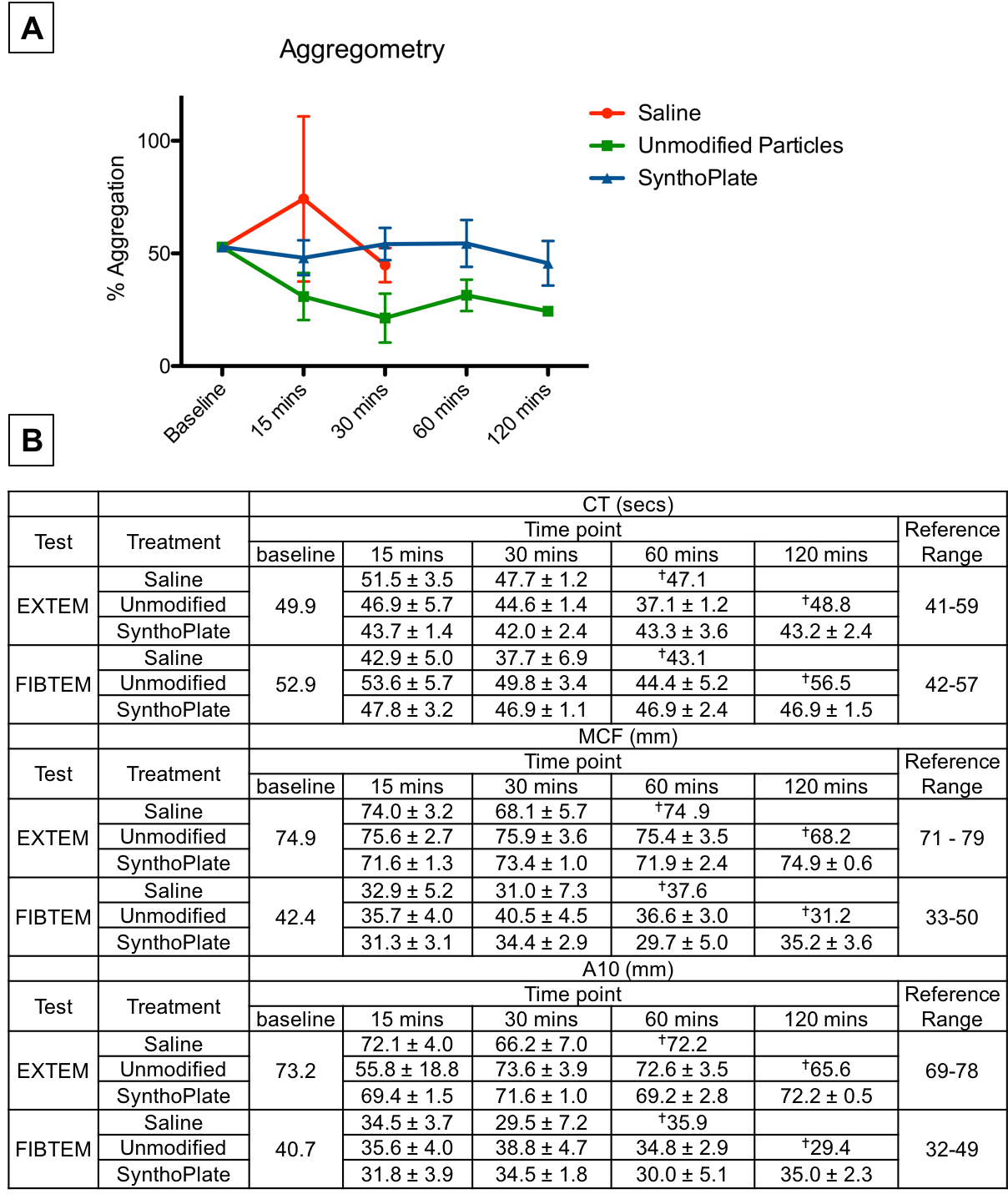

Supplement: Supplementary file 1 — Supplementary Information [file 41598_2018_21384_MOESM1_ESM.docx]
